# Mutant *Samd9l* expression impairs hematopoiesis and induces bone marrow failure in mice

**DOI:** 10.1172/JCI158869

**Published:** 2022-11-01

**Authors:** Sherif Abdelhamed, Melvin E. Thomas, Tamara Westover, Masayuki Umeda, Emily Xiong, Chandra Rolle, Michael P. Walsh, Huiyun Wu, Jason R. Schwartz, Virginia Valentine, Marcus Valentine, Stanley Pounds, Jing Ma, Laura J. Janke, Jeffery M. Klco

**Affiliations:** 1 Department of Pathology and; 2 Department of Biostatistics, St. Jude Children’s Research Hospital, Memphis, Tennessee, USA.; 3 Department of Pediatrics, Vanderbilt University Medical Center, Nashville, Tennessee, USA.; 4 Cytogenetics and; 5 Veterinary Pathology Core, St. Jude Children’s Research Hospital, Memphis, Tennessee, USA.

**Keywords:** Hematology, Hematopoietic stem cells, Mouse models

## Abstract

*SAMD9* and *SAMD9L* germline mutations have recently emerged as a new class of predispositions to pediatric myeloid neoplasms. Patients commonly have impaired hematopoiesis, hypocellular marrows, and a greater risk of developing clonal chromosome 7 deletions leading to MDS and AML. We recently demonstrated that expressing *SAMD9* or *SAMD9L* mutations in hematopoietic cells suppresses their proliferation and induces cell death. Here, we generated a mouse model that conditionally expresses mutant *Samd9l* to assess the in vivo impact on hematopoiesis. Using a range of in vivo and ex vivo assays, we showed that cells with heterozygous *Samd9l* mutations have impaired stemness relative to wild-type counterparts, which was exacerbated by inflammatory stimuli, and ultimately led to bone marrow hypocellularity. Genomic and phenotypic analyses recapitulated many of the hematopoietic cellular phenotypes observed in patients with *SAMD9* or *SAMD9L* mutations, including lymphopenia, and pinpointed TGF-β as a potential targetable pathway. Further, we observed nonrandom genetic deletion of the mutant *Samd9l* locus on mouse chromosome 6, mimicking chromosome 7 deletions observed in patients. Collectively, our study has enhanced our understanding of mutant *Samd9l* hematopoietic phenotypes, emphasized the synergistic role of inflammation in exaggerating the associated hematopoietic defects, and provided insights into potential therapeutic options for patients.

## Graphical abstract

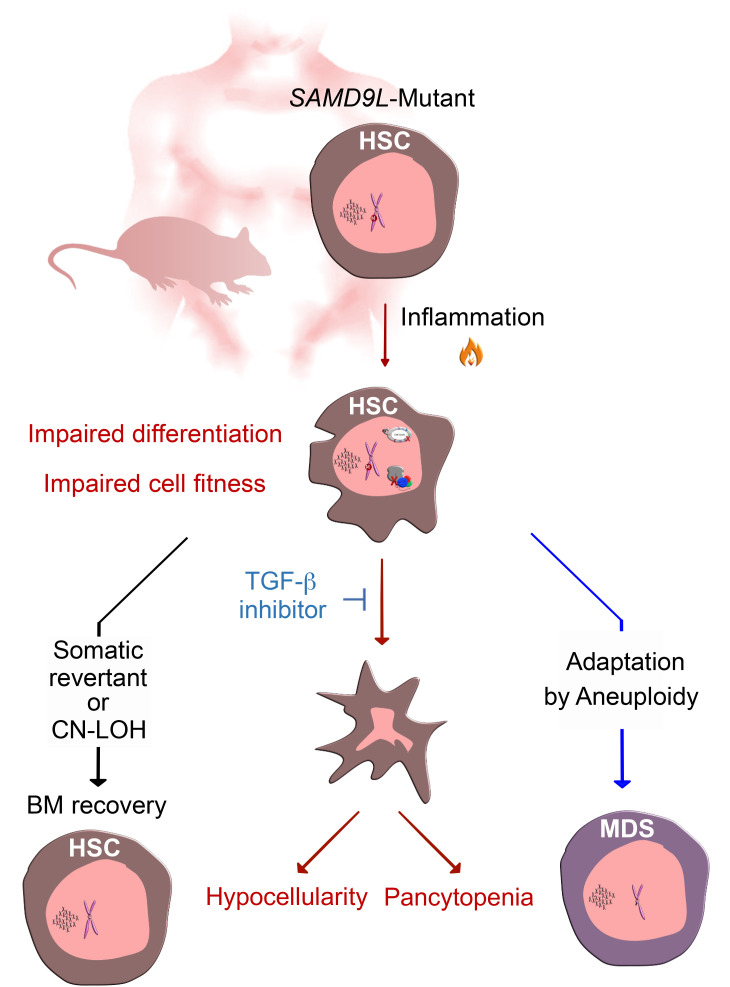

## Introduction

Pediatric myelodysplastic syndromes (MDSs) are characterized by impaired hematopoiesis, peripheral blood (PB) cytopenias, hypocellular bone marrows (BMs), and frequent deletions involving chromosome 7 (chr7) (1) and are associated with unique genetic predispositions (2, 3). Studies from our lab and others identified heterozygous germline mutations in *SAMD9* (sterile α motif domain–containing 9) and its paralog, *SAMD9L*, in 8% to 20% of primary pediatric MDS patients with deletions of chr7 (4, 5). These germline mutations are also associated with transient monosomy 7, BM failure (BMF) syndromes, and acute myeloid leukemia (AML) (6–8), suggesting that these heterozygous germline mutations can have variable impacts on hematopoietic cells.

*SAMD9* and *SAMD9L* are located on human 7q21 and share 60% amino acid homology, while the mouse genome only encodes *Samd9l*, suggesting functional redundancies (9). They are IFN-responsive genes, and their expression dramatically increases following exposure to IFNs and TNF-α (10–12). In addition to the germline mutations observed in patients with BMF and myeloid neoplasms, *SAMD9L* mutations have been associated with inflammatory diseases (13). Conversely, dysregulation of inflammatory pathways, such as TNF-α and IFNs, negatively influence hematopoietic cells and are associated with hematological disorders, including BMF (14–17). We recently highlighted the detrimental effects of overexpressing *SAMD9* and *SAMD9L* mutations ex vivo in primary hematopoietic cells, including the suppression of proliferation and ultimately the induction of cell death (18). However, the impact of *Samd9l* mutations on in vivo hematopoietic cell functional output, fitness, and differentiation from the endogenous IFN-responsive locus has yet to be evaluated.

Here, we describe a conditional knockin mouse model carrying a pathogenic mutation in *Samd9l* that is homologous to a mutation identified in our previously published patient cohort (4). Phenotypic and single-cell analyses of the hematopoietic cells from the *Samd9l*-mutant mouse showed dramatic impairment of cell fitness and lineage composition, leading to a variably penetrant BMF and nonrandom chromosomal loss involving the mutant allele. Inflammatory stimuli upregulated *Samd9l* expression and exacerbated the mutant *Samd9l*–associated hematopoietic phenotypes. We also showed that the TGF-β pathway is activated in *Samd9l*-mutant cells and its inhibition restored their clonogenicity via reverting cell death. These collective data enhance our understanding of pediatric MDS/BMF arising from germline *SAMD9L* mutations and provide insights into potential therapeutic options for this recently described new class of genetic predispositions.

## Results

### Generation of a conditional mutant Samd9l–knockin allele.

**Figure 1:**
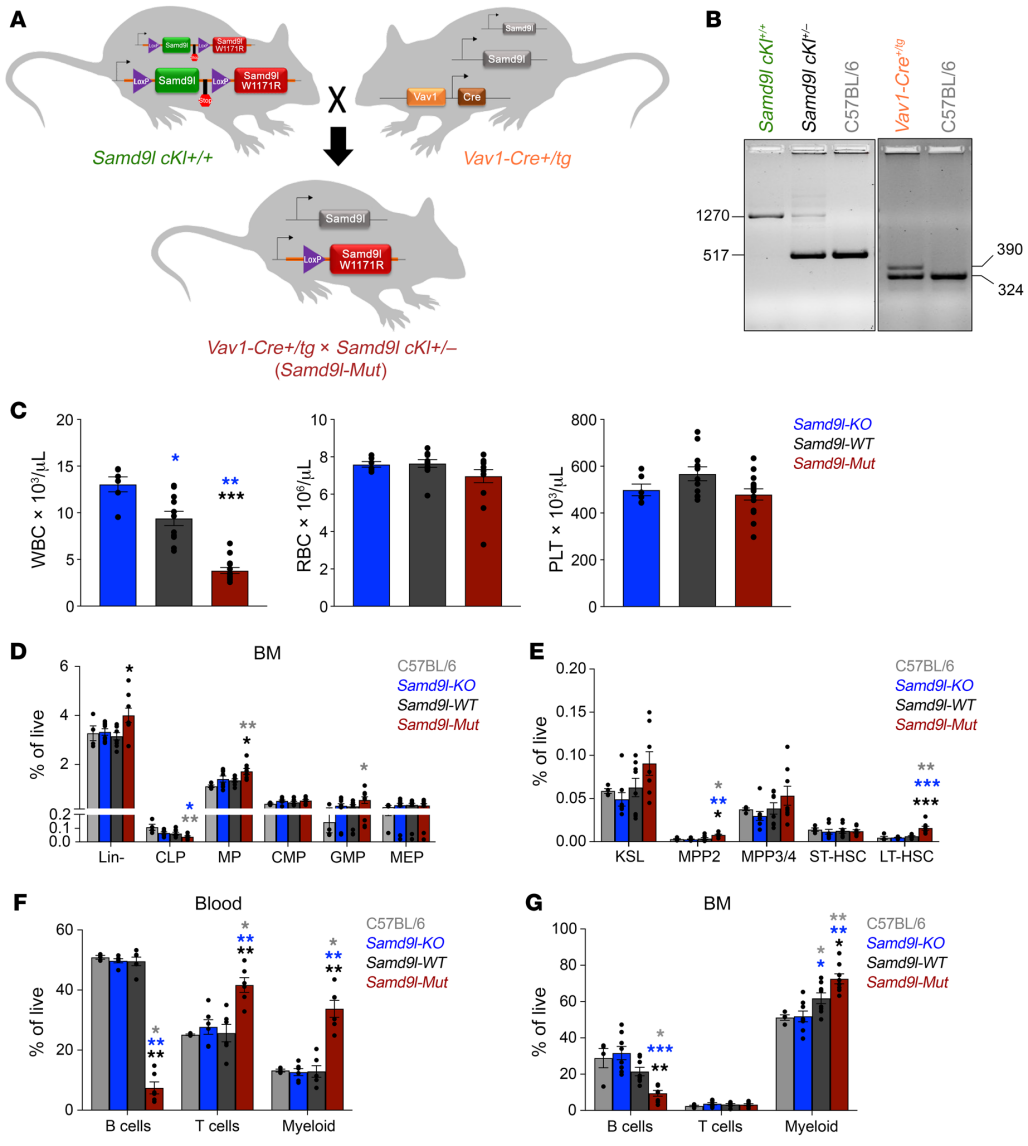
*Samd9l*-*Mut* mouse has altered hematopoiesis. (**A**) Model for the generation of conditional knockin *Samd9l*-W1171R mutation (*Samd9l* cKI^+/+^) crossed with the hematopoietic cell–specific *Vav1*-*Cre* mouse (*Vav1-Cre^+/Tg^*) to produce offspring with heterozygous *Samd9l* mutations (*Vav1-Cre^+/Tg^* *Samd9l cKI^+/–^*). (**B**) PCR analysis verifying the genotypes of the mice. The left gel shows the PCR result for the knockin insertion to have a 1,270-bp product if the cassette is present, 514-bp product if not, and both for heterozygosity. The right gel shows the PCR products for the Vav-1 amplicon using iCre primers (390 bp) and internal positive control (324 bp). (**C**) Complete blood count (CBC) of *Samd9l-KO* (*Samd9l^–/–^*, blue, *n* = 6), *Samd9l-WT* (*Samd9l^cKI+/–^*, black, *n* = 11), and *Samd9l-Mut* (*Vav1*-*Cre^+/Tg^* *Samd9l^cKI+/–^*, red, *n* = 14) mice at 3 months: white blood cells (WBC, left), red blood cells (RBC, middle), and platelets (PLT, right). (**D** and **E**) Flow cytometric analysis of C57BL/6 (gray, *n* = 4), *Samd9l-KO* (blue, *n* = 8), *Samd9l-WT* (black, *n* = 8), and *Samd9l-Mut* (red, *n* = 8) mice assessing the BM compartment for (**D**) Lineage^–^ (Lin^–^), common lymphoid progenitors (CLPs), myeloid progenitors (MPs), common myeloid progenitors (CMPs), granulocyte-macrophage progenitors (GMPs), and megakaryocyte/erythroid progenitors (MEPs), and (**E**) KSL (Lin^–^cKit^+^Sca-1^+^), multipotent progenitors (MPP2 and MPP3/4), and short-term and long-term HSCs (ST-HSCs and LT-HSCs). (**F** and **G**) Percentage of mature cells in (**F**) peripheral blood (PB) or (**G**) BM cells of the C57BL/6, *Samd9l-KO*, *Samd9l-WT*, and *Samd9l-Mut* mice assessed by flow cytometry. For panels **C**–**G**, groups were initially compared by Kruskal-Wallis test. Significant Kruskal-Wallis results were followed by pairwise comparisons with Wilcoxon’s rank-sum test. *P < 0.05; **P < 0.01; ***P < 0.001. Error bars indicate the SEM for biological replicates.

We generated a conditional knockin mouse model carrying the *Samd9l*-W1171R point mutation, corresponding to the human W1180R mutation previously reported by our group (4) to study the in vivo impact of *Samd9l* mutations. Gene targeting was used to introduce LoxP sites flanking a GFP-fused *Samd9l-WT* exon 2 (the first coding exon) followed by an FRT-flanked neomycin selection cassette upstream of a stop codon and an mCherry-fused mutant *Samd9l* exon 2 containing the W1171R mutation (Supplemental Figure 1A; supplemental material available online with this article; https://doi.org/10.1172/JCI158869DS1). Following removal of the neomycin cassette, the generated *Samd9l^fl/fl^* (*Samd9l^cKI+/+^*) mice were bred with transgenic mice containing the hematopoietic cell–specific *Vav1*-*Cre* to generate a heterozygous mutation (which is universally observed in patients) in the hematopoietic compartment (Supplemental Figure 1, B and C). These *Vav1-Cre^+/Tg^* *Samd9l*^cKI+/–^ mice (*Samd9l-Mut*) are viable and fertile with offspring born at the expected Mendelian ratios (Figure 1, A and B). Of note, attempts to induce the mutation throughout the animal early in development by crossing with *CMV*-*Cre* mice led to a lack of viable offspring. Three-month-old *Samd9l-Mut* mice demonstrated a distinct reduction in white blood cell (WBC) counts, with no apparent difference in red blood cell (RBC) and platelet counts (Figure 1C) when compared to the *Samd9l*^cKI+/–^ mice (*Samd9l-WT*) and the previously reported *Samd9l^–/–^* mice (*Samd9l-KO*) (19).

### Samd9l-mutant hematopoiesis favors myeloid commitment.

We next compared the BM compartments of 3-month-old *Samd9l-Mut* mice with *Samd9l-KO*, *Samd9l-WT*, and native C57BL/6 mice and observed a significant relative increase in the percentage of Lin^–^cKit^+^ myeloid progenitors (MPs), with a decrease in the lymphoid lines, including the common lymphoid progenitor (CLP; Lin^–^cKit^lo^Sca-1^lo^CD127^+^) (Figure 1D and Supplemental Figure 1, D and E). We also observed an increase in granulocytic/monocytic progenitors (GMPs). Within the hematopoietic stem and progenitor cell (HSPC) compartment, both the MPP2 population (Lin^–^cKit^+^Sca-1^+^CD48^+^CD150^+^, the precursors for myeloid cells) and the long-term HSCs (LT-HSCs; Lin^–^cKit^+^Sca-1^+^CD48^–^CD150^+^) were increased in the *Samd9l-Mut* mice (Figure 1E). Notably, lymphoid progenitors had lower percentages of EdU incorporation in *Samd9l-Mut* BM cells relative to other groups, whereas MP and KSL (Lin^–^cKit^+^Sca-1^+^) populations had higher relative percentages (Supplemental Figure 1F). This skewing was further noted in the mature cells, as we observed a significant decrease in B cells accompanied by a relative increase in the percentage of the mature myeloid cells in the PB, BM, and spleen (Figure 1, F and G, and Supplemental Figure 1, G and H). This effect was pronounced in B cells in *Samd9l-Mut* mice, which also showed a lower EdU incorporation percentage of the total proliferating cells relative to myeloid and T cells (Supplemental Figure 1I). We then wanted to examine whether the basal expression level of the *Samd9l* gene in different cell types could correlate to their diverse sensitivity to the mutations. To test this, we analyzed publicly available data sets and observed a higher *Samd9l* level in B cells relative to other cell types in humans or mice, which we also confirmed by qPCR analysis of sorted murine cells (Supplemental Figure 2, A–C). Moreover, *Samd9l* expression was directly proportional to B cell maturation (Supplemental Figure 2D). Notably, we utilized a well-established flow cytometric assessment of B cell maturation stages (20) and demonstrated a clear suppression of the mature B cell populations in *Samd9l-Mut* mice relative to *Samd9l-WT* mice, whereas the less mature cells were not affected (Supplemental Figure 2, E and F). Collectively, these findings indicate that *Samd9l-Mut* expression affects all levels of the hematopoietic hierarchy, with the most significant impact on mature B cells, likely due to their elevated levels of *Samd9l* expression.

**Figure 2:**
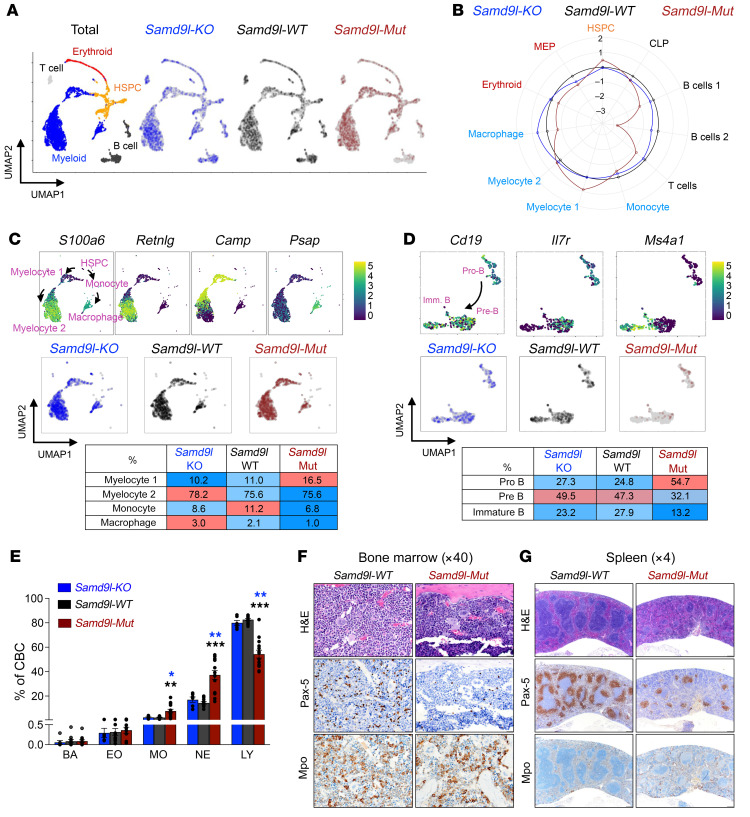
Mutant *Samd9l* expression skews murine hematopoietic lineage profiles. (**A** and **B**) scRNA-seq of *Samd9l-KO* (*n* = 1,495), *Samd9l-WT* (*n* = 1,498), and *Samd9l*-*Mut* (*n* = 1,436) mice from a single experiment. (**A**) Uniform manifold approximation and projection (UMAP) plots of scRNA-seq data. Eleven clusters were identified according to established marker expression and consolidated into 5 main populations as annotated. (**B**) A circular plot showing the proportion of the identified 11 clusters in each group relative to *Samd9l-WT* control. (**C**) Myeloid cell differentiation trajectories based on reported markers, including *S100a6*, *Retnig*, *Camp*, *Psap*, and others on the scRNA-seq UMAP plot (top panel) and the percentage of cells in each cluster (middle and bottom panels) from *Samd9l-KO*, *Samd9l-WT*, and *Samd9l-Mut* mice. The colors of each dot represent the normalized expression level of genes indicated above. (**D**) B cell differentiation trajectory based on established markers, including *Cd19*, *Il7r*, and *Ms4a1* (encodes CD20 protein) (top panel) and the percentage of cells in each cluster (middle and bottom panels) from *Samd9l-KO*, *Samd9l-WT*, and *Samd9l-Mut* mice. (**E**) CBC of *Samd9l-KO* (*n* = 6), *Samd9l-WT* (*n* = 11), and *Samd9l-Mut* (*n* = 14) mice at 3 months of age showing neutrophils (NEs), monocytes (MOs), eosinophils (EOs), basophils (BAs), and lymphocytes (LYs). (**F** and **G**) Histological assessment of BM (**F**, ×40 magnification) and spleen (**G**, ×4 magnification) sections from 3-month-old *Samd9l-WT* or *Samd9l-Mut*. Sections were stained with modified Romanowsky stain for morphological assessment and IHC labeling was done using anti–PAX-5 (B cells) or anti-MPO (myeloid cells). For panel **E**, groups were initially compared by Kruskal-Wallis test. Significant Kruskal-Wallis results were followed by pairwise comparisons with Wilcoxon’s rank-sum test. *P < 0.05, **P < 0.01, ***P < 0.001. Error bars indicate the SEM for biological replicates.

Single-cell transcriptomics was then used to further study the BM compartments of these mice. Using established lineage markers (21, 22) (Supplemental Figure 3A), 11 cell clusters were identified, and further consolidated into 5 main populations (myeloid, B cells, T cells, erythroid, and HSPCs) (Figure 2A). As noted by flow cytometry, the *Samd9l-Mut* have a clear decrease in B cell populations, an increase in myeloid cells, and additional alterations in the HSPC population (Figure 2B and Supplemental Figure 3, B–D). The HSPC subcompartments were further defined by the expression of *Cd34*, *Car1*, *Mpo*, *Fn1*, and *Il7* (Supplemental Figure 3E) using single-cell RNA sequencing (scRNA-seq). Differentially expressed gene (DEG) analysis of the HSPC population showed enrichment of genes involved in translation and cell division in the *Samd9l-Mut* relative to *Samd9l-WT* mice (Supplemental Figure 3F). Of the myeloid cell populations, *Samd9l-Mut* mice had a relative increase in MPs and a decrease in monocytes and macrophages, along with expression changes associated with cell cycle regulation and immune response (Figure 2C and Supplemental Figure 3G). Moreover, *Samd9l-Mut* mice exhibited a relative increase in the less differentiated B cell population (Pro-B) and a relative decrease in the more mature populations (Pre-B and immature B) (Figure 2D). Notably, we observed an enrichment of genes related to translation, apoptosis, and DNA replication in B cells in *Samd9l-Mut* (Supplemental Figure 3H). Similarly, complete blood counts (CBCs) from these mice demonstrated global lymphopenia with a relative increase in the percentage of myeloid cells (Figure 2E). These changes in the mature cells were supported by immunohistochemistry (IHC) on BM and spleen sections, which showed a relative decrease in B cells (stained by anti–PAX-5) and a slight increase in myeloid precursors (stained by anti-MPO) in *Samd9l-Mut* versus *Samd9l-WT* (Figure 2, F and G). Together, these scRNA-seq data confirm the global hematopoietic lineage alterations observed by flow cytometry and further highlight the clear deterioration of B cells.

### Samd9l mutation impairs hematopoietic cell fitness and repopulation potential.

**Figure 3:**
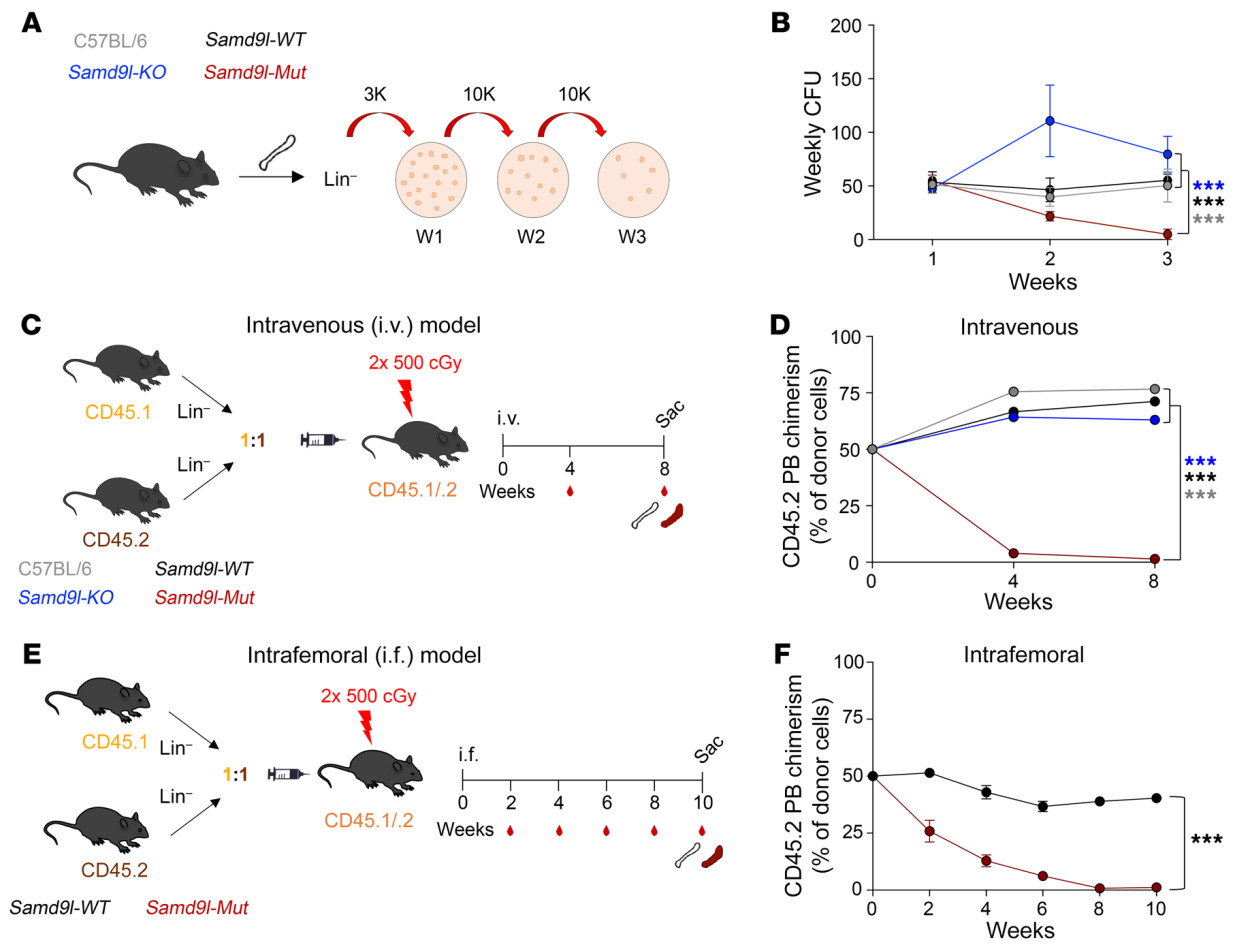
Impaired ex vivo and in vivo fitness of *Samd9l*-mutant cells. (**A** and **B**) Serial colony-forming unit cell (CFU-C) analysis of Lin^–^ cells from C57BL/6, *Samd9l-KO*, *Samd9l-WT*, and *Samd9l-Mut* mice. (**A**) Cells were plated (3,000 cells/plate) in methylcellulose media. One week later, colonies were counted and serially replated (10,000 cells/plate) for a total of 3 rounds. (**B**) The number of colonies in the weekly replates. (**C**) Schematic illustration of the competitive transplantation model. Lin^–^ cells from CD45.2 (C57BL/6, *Samd9l-KO*, *Samd9l-WT*, or *Samd9l-Mut* cells) and CD45.1 (wild-type competitor) were injected i.v. at a 1:1 ratio via tail vein into lethally irradiated CD45.1/CD45.2 mice. (**D**) CD45.2 chimerism in the PB of recipient animals (*n* ≥ 12 per group) from 0 to 8 weeks. (**E**) Intrafemoral (i.f.) competitive transplantation scheme. Lin^–^ cells from CD45.2 (*Samd9l-WT* or *Samd9l-Mut* cells) and CD45.1 (wild-type competitor) were injected at a 1:1 ratio into the femurs of lethally irradiated CD45.1/CD45.2 mice. (**F**) CD45.2 chimerism in the PB of recipient animals in **E** (*n* ≥ 8 per group). Time is denoted in weeks after injections. A longitudinal mixed-effects regression model was used for statistical analysis. Initially, a global test of whether all 4 groups had the same longitudinal trend was performed. A significant result from this global test was followed by pairwise tests evaluating the equality of trends over time for the 2 groups. ***P < 0.001. Error bars indicate the SEM for biological replicates. Gray, C57BL/6; blue, *Samd9l-KO*, black, *Samd9l-WT*; red, *Samd9l-Mut*.

Next, we assessed the functional output of *Samd9l*-mutant cells and their relative hematopoietic fitness. We first examined the serial colony formation of BM cells from *Samd9l-Mut* mice as a correlate of self-renewal potential (Figure 3A). Relative to wild-type *Samd9l* controls, *Samd9l-Mut* cells exhibited a gradual decrease in clonogenicity that almost completely diminished at the third replating (Figure 3B). *Samd9l-KO* cells showed a mildly stronger replating potential and a greater number of cells per colony than wild-type controls (Figure 3B and Supplemental Figure 4A), reminiscent of the reported repopulation advantage resulting from *Samd9l* haploinsufficiency (19). We observed no apparent differences in colony subtypes (Supplemental Figure 4B). Next, we transplanted Lin^–^ BM cells from CD45.2 mice (C57BL/6, *Samd9l-WT*, *Samd9l-KO*, or *Samd9l-Mut*) mixed in a 1:1 ratio with age-matched CD45.1 competitor cells and injected via tail-vein intravenous (i.v.) injections into lethally irradiated heterozygous CD45.1/CD45.2 recipient mice to examine in vivo competitive fitness (Figure 3C). Among the compared groups, only *Samd9l-Mut* cells were significantly outcompeted by wild-type CD45.1 cells (Figure 3D). This phenotype was sustained even at 16 weeks after transplantation (data not shown). Upon gating on the CD45.2 populations, we observed a proportional increase in myeloid cells at the expense of lymphoid cells in the *Samd9l-Mut* relative to other groups (Supplemental Figure 4C). To determine whether the competitive disadvantage of *Samd9l-Mut* cells resulted from impaired stem cell fitness and not a failure of homing to the BM, we performed a competitive transplantation experiment via intrafemoral injections (Figure 3E). Consistent with the i.v. data, weekly PB analysis showed a gradual competitive disadvantage of *Samd9l-Mut* cells, but not the *Samd9l-WT* cells (Figure 3F). We also observed similar patterns in the BM and spleen at sacrifice (Supplemental Figure 4D). After the adoptive transfer into irradiated recipients, the *Samd9l-Mut* donor compartment showed a relative increase in the percentage of myeloid cells, with a decrease in B cells in comparison with *Samd9l-WT* donors (Supplemental Figure 4, E–G), which mirrors the impacts shown in Figure 1. Altogether, *Samd9l*-mutant cells demonstrated a profound lack of fitness and repopulation capacities ex vivo and in vivo.

### Inflammation upregulates mutant Samd9l and further potentiates its pathogenicity.

**Figure 4:**
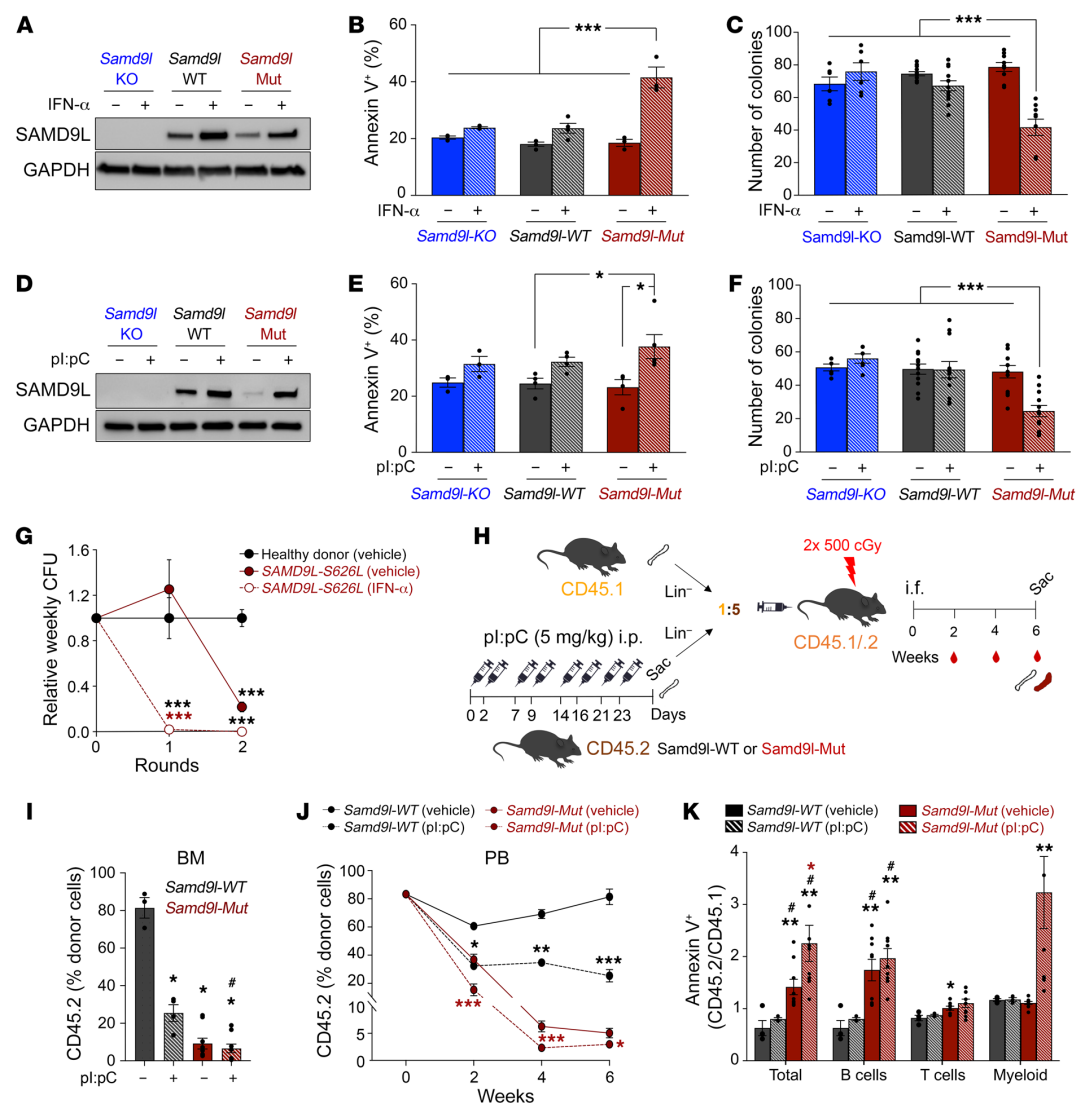
Inflammation promotes *Samd9l*-mutant cell death and further impairs repopulation potential. (**A**–**C**) SAMD9L protein expression (**A**), annexin V^+^ cell percentage (**B**), and CFU-C (**C**) of BM harvested from indicated mice and treated twice with IFN-α (1,000 U) or vehicle for 48 hours ex vivo. For CFU-C, BM cells (10,000) were cultured in methylcellulose media, and colonies were counted after 1 week. (**D**–**F**) SAMD9L protein expression (**D**), annexin V^+^ cell percentage (**E**), and CFU-C (**F**) of BM harvested from indicated mice treated with pI:pC (5 mg/kg) or vehicle twice a week for 4 weeks. (**G**) Weekly colony counts of vehicle- or IFN-α–treated (1,000 U) BM cells derived from a patient harboring the *SAMD9L*-S626L mutation normalized to healthy donor cord blood. (**H**) Model for *Samd9l-WT* and *Samd9l-Mut* pI:pC treatment followed by 5:1 competitive intrafemoral BM transplants of CD45.2 (*Samd9l-WT* or *Samd9l-Mut* treated with pI:pC or vehicle) versus CD45.1. (**I**–**K**) CD45.2 chimerism in BM at 6-week endpoint (**I**) and PB biweekly (**J**). The comparison was done between *Samd9l-Mut* vehicle (*n* = 9) vs. *Samd9l-Mut* pI:pC (*n* = 9) or *Samd9l-WT* vehicle (*n* = 3) vs. *Samd9l-WT* pI:pC (*n* = 4). (**K**) Relative levels of annexin V^+^ cells within total BM, B, T, or myeloid cells of the donor cells from each group (ratio of annexin V^+^ CD45.2 divided by annexin V^+^ CD45.1 cells. For panels **G** and **J**, a longitudinal mixed-effects regression model was used for statistical analysis followed by pairwise Tukey-adjusted tests. For panel **K**, the data were not normally distributed; therefore, the Kruskal-Wallis test was initially performed and followed by Wilcoxon’s rank-sum test for pairwise comparisons. For all other panels, 2-way ANOVA was used to evaluate the main effects and genotype/treatment interactions and followed by Tukey’s pairwise comparisons. Error bars indicate the SEM for biological replicates. Blue, *Samd9l-KO*, black, *Samd9l-WT*; red, *Samd9l-Mut*. *P < 0.05; **P < 0.01; ***P < 0.001 compared with vehicle-treated groups. ^#^*P* < 0.05 compared with pI:pC-treated groups. Color indicates the comparison group.

Considering that *Samd9l* is a known IFN-responsive gene (10), we next addressed the impact of inflammation-induced upregulation of *Samd9l-Mut* expression on hematopoietic cell function. We confirmed an increase in *SAMD9* and *SAMD9L* RNA and protein levels after IFN-α treatment in HEK293T and human cord-blood CD34^+^ cells (Supplemental Figure 5, A–C). Similarly, IFN-α ex vivo treatment increased SAMD9L expression in BM cells isolated from *Samd9l-WT* and *Samd9l-Mut* but not those from *Samd9l-KO* (Figure 4A). IFN-α significantly decreased *Samd9l-Mut* cell growth and viability, which was associated with a marked increase in cell death (Figure 4B and Supplemental Figure 5D), and a significant decrease in clonogenicity of the *Samd9l-Mut* cells (Figure 4C). We also observed a significant decrease in cell cycle (EdU incorporation) and translation (O-propargyl-puromycin incorporation) in *Samd9l-Mut* after IFN-α (Supplemental Figure 5, E and F), consistent with our previous in vitro studies (18). Next, we utilized polyinosinic/polycytidylic acid (pI:pC), a Toll-like receptor 3 agonist, to induce type-I IFNs in vivo. We treated *Samd9l-WT*, *Samd9l-Mut*, and *Samd9l-KO* mice with 5 mg/kg pI:pC twice a week for 4 weeks (23, 24). Similar to IFN-α, pI:pC increased SAMD9L protein expression in both *Samd9l-WT* and *Samd9l-Mut* mice (Figure 4D), with a resulting significant reduction in colony formation and induction of apoptosis in BM cells from *Samd9l-Mut* (Figure 4, E and F). Likewise, BM cells isolated from a patient harboring the *SAMD9L*-S626L mutation treated with IFN-α exhibited a complete loss in colony formation relative to vehicle-treated cells or control cord-blood healthy donor cells (Figure 4G). Our data collectively demonstrate that inflammatory stress mediates an increase in *Samd9l* expression, ultimately enhancing the cellular phenotypes associated with the expression of a mutant allele.

### Inflammatory stimulus further impairs hematopoietic cell fitness.

Next, we assessed the in vivo impact of inflammation on hematopoietic cell fitness using a 5:1 competitive BM transplantation model of *Samd9l-WT* or *Samd9l-Mut* mice (CD45.2) pretreated with pI:pC or vehicle (Figure 4H). Despite the initial higher ratio of input CD45.2 cells at injection, *Samd9l-Mut* groups were outcompeted by CD45.1 cells. Notably, we observed a further competitive disadvantage of pI:pC-treated *Samd9l-Mut* cells in comparison with vehicle-treated *Samd9l-Mut* (Figure 4, I and J, and Supplemental Figure 5G). Similarly, pI:pC decreased *Samd9l-WT* fitness relative to vehicle-treated cells (Figure 4, I and J, and Supplemental Figure 5G). We observed no significant difference in mature cell composition in both *Samd9l-Mut* and *Samd9l-WT* treated with pI:pC relative to vehicle (Supplemental Figure 5H). Importantly, while vehicle-treated *Samd9l-Mut* donor cells had significant increases in B cell apoptosis, pI:pC-treated mice had significant increases in apoptosis for B and myeloid cells as a result of the combined transplant and inflammatory stresses (Figure 4K). Similarly, 1:1 competitive transplantation also showed lowered *Samd9l-Mut* fitness with inflammation (Supplemental Figure 5, I–K).

### The lack of fitness in Samd9l-mutant cells is partly mediated by TGF-β activation.

**Figure 5:**
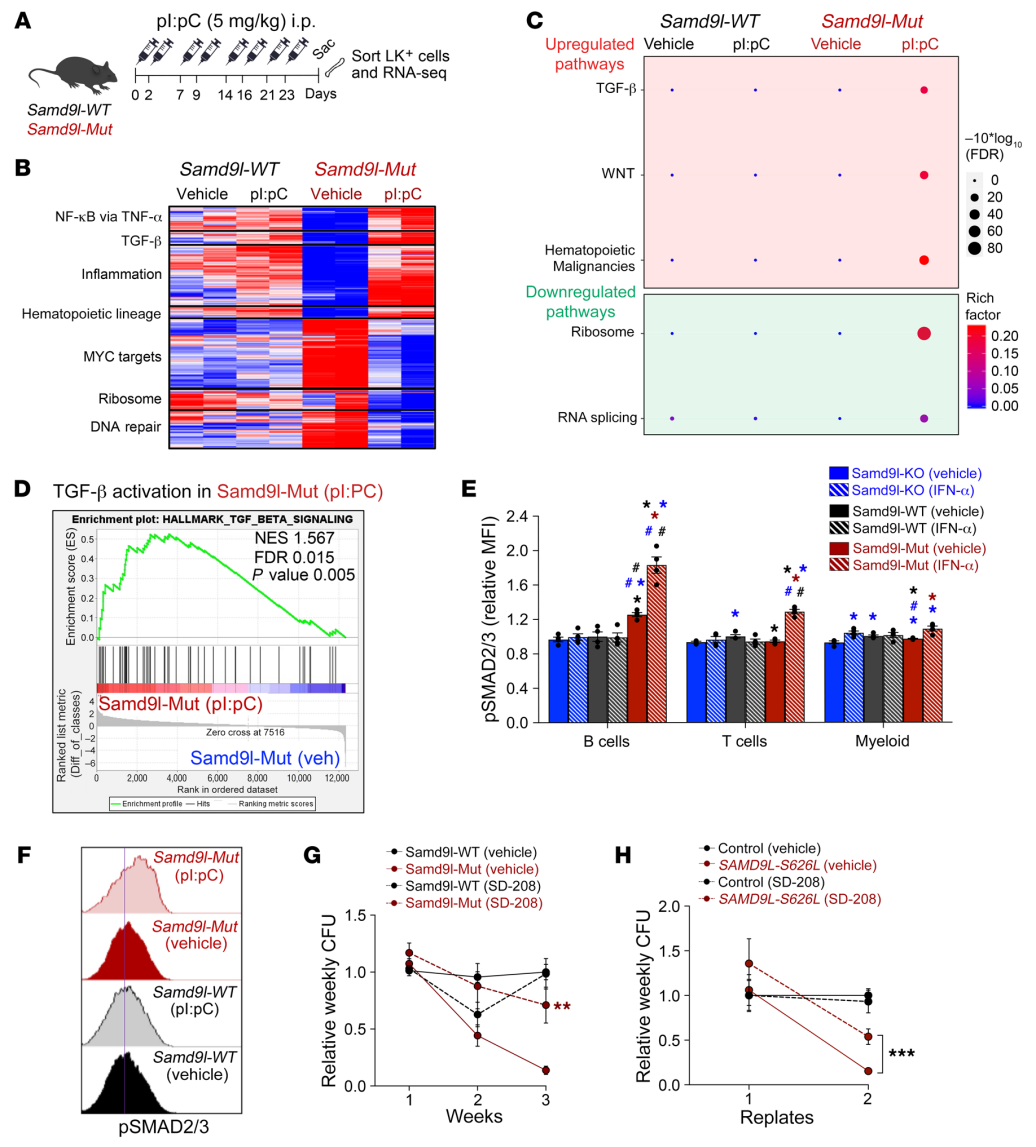
The lack of fitness in *Samd9l*-mutant cells is partly via TGF-β activation. (**A**) Model of pI:pC treatment regimen followed by sorting the Lin^–^cKit^+^ (LK) population from *Samd9l-WT* and *Samd9l-Mut* mice to perform RNA-seq. (**B**) Heatmap showing the up- and downregulated pathways in *Samd9l-WT* and *Samd9l-Mut* treated with vehicle or pI:pC. (**C**) A plot of pathway enrichments of DEGs downregulated in pI:pC-treated *Samd9l-Mut* mice relative to vehicle-treated. The size of the circles represents gene counts, and the significance was determined by FDR. The color is dependent on the fold of enrichment. (**D**) Gene set enrichment analysis (GSEA) showing TGF-β pathway activation in pI:pC-treated *Samd9l-Mut* mice relative to vehicle-treated *Samd9l-Mut* mice. Normalized enrichment score (NES), FDR, and *P* value are indicated. (**E**) Phospho-SMAD2/3 expression in *Samd9l-KO*, *Samd9l-WT*, and *Samd9l-Mut* BM cells treated with IFN-α or vehicle (*n* = 4 per group). (**F**) Representative histograms of phospho-SMAD2/3 expression in B cells of *Samd9l-WT* and *Samd9l-Mut* cells after IFN-α or vehicle. (**G** and **H**) Serial CFU-C replating of *Samd9l-WT* and *Samd9l-Mut* cells (**G**) or human cells from a patient with *SAMD9L*-S626L mutation or control (**H**) with or without TGF-β inhibitor (SD-208). Data show at least 3 independent experiments. For panel **E**, Kruskal-Wallis test was used to perform an initial comparison across all groups and followed by pairwise comparisons with Wilcoxon’s rank-sum test. For panel **G**, for each genotype/time point, we used Wilcoxon’s rank-sum test to compare across the 2 treatments because data were not normally distributed. For panel **H**, a longitudinal mixed-effects regression model was used for statistical analysis followed by pairwise Tukey-adjusted tests evaluating the equality of means across each pair of groups at each time point. *P < 0.05, **P < 0.01, ***P < 0.001 compared with vehicle-treated groups. ^#^*P* < 0.05 compared with pI:pC-treated groups. Error bars indicate the SEM for biological replicates. Blue, *Samd9l-KO*; black, *Samd9l-WT*; red, *Samd9l-Mut* (red); stripes, IFN-α or pI:pC; solid, vehicle. Color indicates the comparison group.

We next performed RNA-seq analysis of Lin^–^cKit^+^ HSPCs from *Samd9l-WT* or *Samd9l-Mut* mice with or without pI:pC treatment and found a variety of upregulated pathways (including inflammatory signaling such as TNF-α/NF-κB and JAK/STAT signaling and hematopoietic lineage) and downregulated pathways (MYC, ribosome, and DNA repair) (Figure 5, A–C, and Supplemental Figure 6, A and B) (18). Importantly, the data revealed an upregulation of genes involved in TGF-β pathways in *Samd9l-Mut* mice treated with pI:pC relative to all other groups (Figure 5, B and C, and Supplemental Figure 6, B and C). Gene set enrichment analysis (GSEA) confirmed the activation of genes involved in the TGF-β pathway in *Samd9l-Mut* cells in response to pI:pC (Figure 5D). We also found a consistent TGF-β enrichment in hCD34^+^ cells with *SAMD9L*-W1180R overexpression (Supplemental Figure 7A). Mechanistically, TGF-β transmits intracellular signals through the SMAD family and its activity is measured by SMAD2/3 phosphorylation (25). Intracellular phospho-flow revealed hyperphosphorylation of SMAD2/3 in total BM cells from *Samd9l-Mut* after IFN-α or pI:pC treatment (Supplemental Figure 7, B and C). Among BM cells, SMAD2/3 activation was more obvious in the B cell population of *Samd9l-Mut* in response to inflammatory stimuli (Figure 5, E and F, and Supplemental Figure 7D). Consistent with previous reports on other BM disorders (25, 26), treatment with SD-208, a small-molecule TGF-β inhibitor, rescued the mutation-dependent reduction in clonogenicity and reverted the apoptotic phenotype of *Samd9l-Mut* cells, with no effect on *Samd9l-WT* cells (Figure 5G and Supplemental Figure 7E). Similarly, SD-208 improved the impaired clonogenicity in BM cells isolated from a patient with the *SAMD9L*-S626L mutation (Figure 5H).

### Inflammation augments B cell lymphopenia and impairs erythroid maturation.

**Figure 6:**
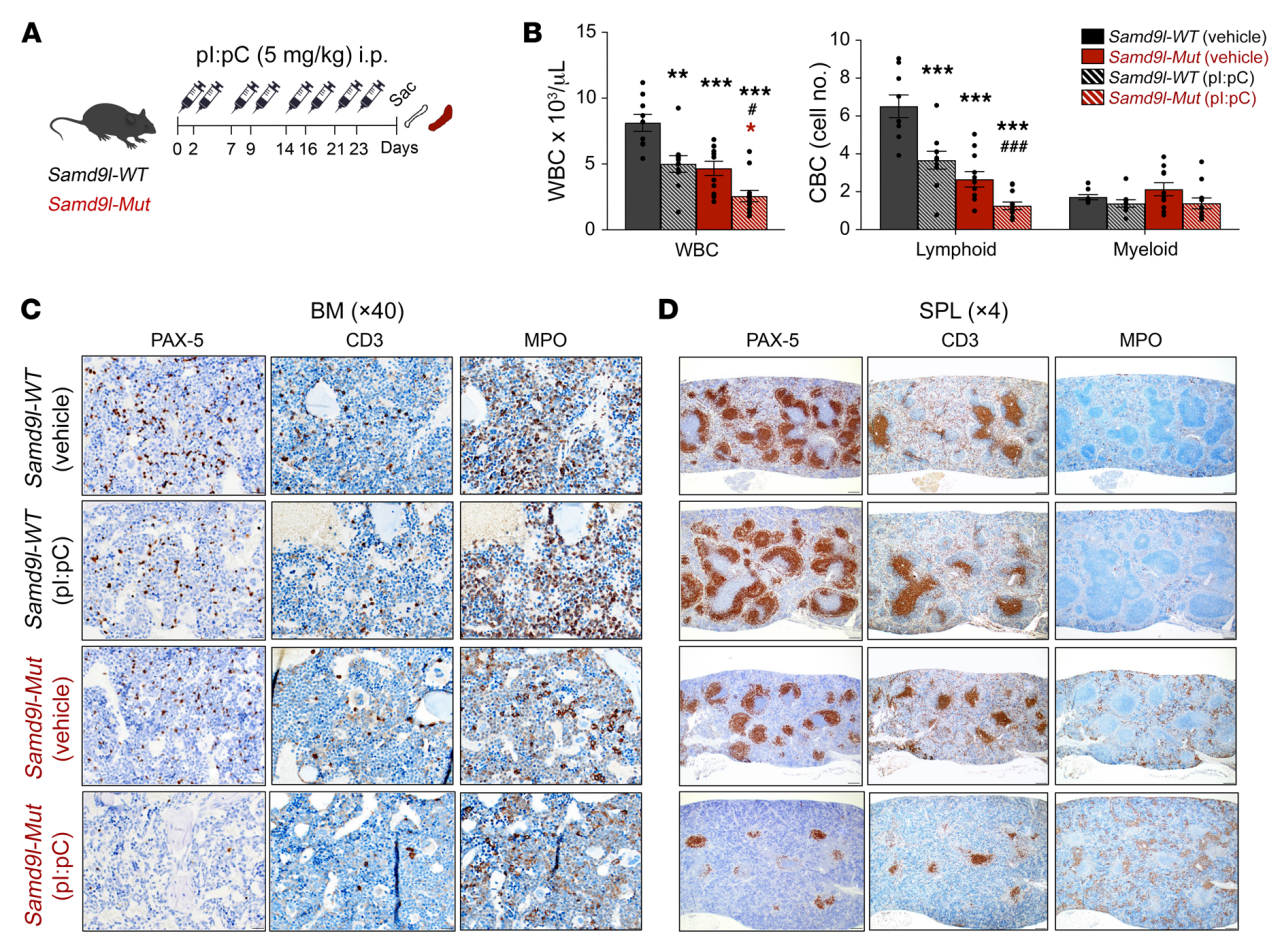
Inflammation worsens cytopenias in *Samd9l*-*Mut* mouse. (**A**) Illustration for treatment of *Samd9l-WT* and *Samd9l-Mut* mice with pI:pC or vehicle. (**B**) CBC analysis showing WBC, lymphoid cells, and monocytes/neutrophils/basophils (myeloid) for the treated mice (*n* = 8). (**C** and **D**) BM (**C**) and spleen (**D**) were stained with anti–PAX-5, anti-CD3, and anti-MPO to assess B, T, and myeloid cells, respectively (*n* = 2 per group). For panel **B**, the Kruskal-Wallis test was used to perform an initial comparison across all groups, and followed by pairwise comparisons with Wilcoxon’s rank-sum test. *P < 0.05, **P < 0.01, ***P < 0.001 compared with vehicle-treated groups. ^#^*P* < 0.05, ^###^*P* < 0.001 compared with pI:pC-treated groups. Color indicates the comparison group. Error bars indicate the SEM for biological replicates. Black, *Samd9l-WT*, red, *Samd9l-Mut*; stripes, pI:pC; solid, vehicle.

We next treated *Samd9l-Mut* and *Samd9l-WT* mice with pI:pC to assess the impact of inflammatory stimuli on the hematopoietic compartments in vivo (Figure 6A). PB counts, particularly lymphocytes, were decreased after pI:pC treatment in *Samd9l-Mut* and *Samd9l-WT* mice; however, there were no changes in myeloid cells (Figure 6B). Morphologic evaluation of BM revealed predominantly mature neutrophils, with scattered lymphocytes, myeloblasts, and erythroblasts in *Samd9l-WT* mice, as expected, regardless of whether they were treated with vehicle or pI:pC (Supplemental Figure 8A). In contrast, *Samd9l-Mut* mice had a higher abundance of myeloblasts and fewer lymphocytes relative to *Samd9l-WT* groups, and these effects were remarkably exaggerated with pI:pC, with no significant dysplasia (Supplemental Table 1). Histological examination of BM demonstrated no architectural changes; however, a clear distortion of splenic architecture in the pI:pC-treated *Samd9l-Mut* mice was observed (Supplemental Figure 8, B and C). Additionally, thymuses from *Samd9l-Mut* treated with pI:pC exhibited either cortical apoptosis with tingible body macrophages or atypical hyperplasia (Supplemental Figure 8D). Importantly, IHC studies confirmed the reduction in lymphocytes and moderate myeloid hyperplasia in pI:pC-treated *Samd9l-Mut* mice compared with all other groups (Figure 6, C and D).

**Figure 7:**
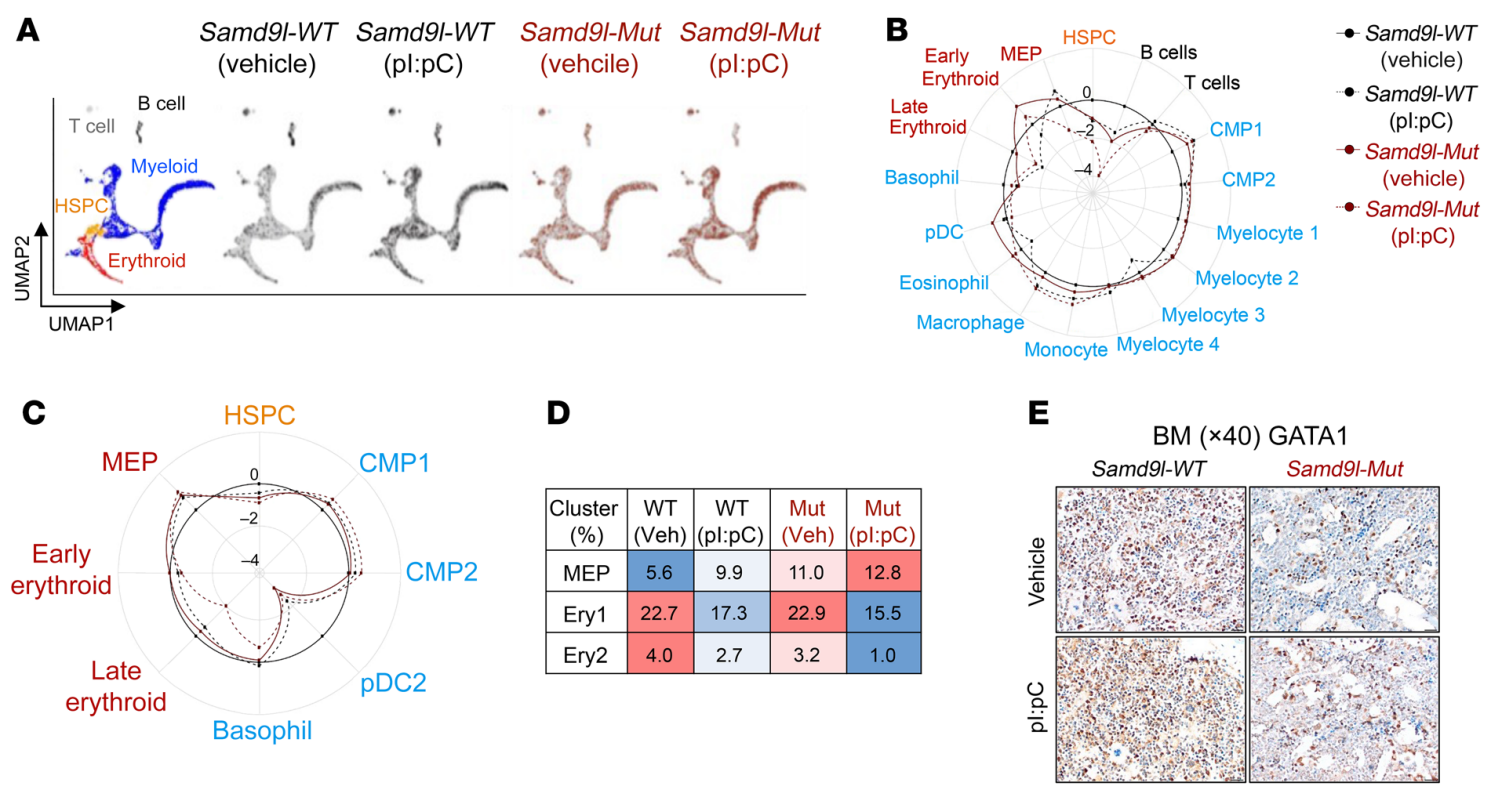
Inflammation impairs cell maturation in *Samd9l*-*Mut* mouse. (**A**–**D**) scRNA-seq of WBM and Lin^–^cKit^+^ (LK) sorted cells from *Samd9l-WT* or *Samd9l-Mut* mice treated with either pI:pC or vehicle. (**A**) UMAP plots of scRNA-seq data showing 5 main populations. (**B**) A circular plot showing the proportion of the identified 17 clusters from each WBM sample relative to WBM from *Samd9l-WT* control. (**C**) A circular plot showing 8 clusters from LK sorted samples. (**D**) A heatmap of the proportion of cells in each erythroid maturation stage is denoted as megakaryocyte/erythroid progenitor (MEP), early erythroid (Ery 1), and late erythroid (Ery 2). Among the compared groups, red indicates higher levels and blue indicates lower levels, and values represent the percentage of total cells. (**E**) BM cross sections (magnification, ×40) from *Samd9l-WT* or *Samd9l-Mut* mice treated with pI:pC or vehicle stained with anti-GATA1 antibody.

We next performed scRNA-seq on both whole BM (WBM) and sorted Lin^–^cKit^+^ HSPCs from *Samd9l-WT* and *Samd9l-Mut* mice with or without pI:pC (Supplemental Figure 9, A–C). Among the WBM populations, consistent with Figure 2, A and B, we observed a significant decrease in B cells and a slight increase in the percentage of the myeloid populations in the *Samd9l-Mut* mice, which were further exacerbated with pI:pC (Figure 7, A and B, and Supplemental Figure 9, C–E). DEGs of the HSPC population included enriched genes involved in DNA replication, translation, and response to viral genes in the pI:pC-treated *Samd9l-Mut* mouse relative to the vehicle-treated mouse (Supplemental Figure 9E). Myeloid differentiation trajectory supported our previous observation of the increased percentage of MPs in *Samd9l-Mut*, which appear more pronounced with pI:pC (Supplemental Figure 9F). B cell maturation trajectory analysis showed a decrease in the relatively mature cells in the BM (immature-B) in *Samd9l-Mut* versus *Samd9l-WT*. Treatment with pI:pC almost abolished B cells at all stages, likely due to cellular damage indicated by the increased proapoptotic and inflammatory markers and decreased proliferation markers (Supplemental Figure 9, G and H). Our single-cell data from Lin^–^cKit^+^ cells showed a remarkable decrease in the more mature erythroid progenitors in *Samd9l-Mut* after pI:pC (Figure 7, C and D). Correspondingly, staining of BM sections using anti-GATA1 or anti-CD235a showed a distinct decrease in erythroid cells and an increased myeloid/erythroid ratio in the pI:pC-treated *Samd9l-Mut* (Figure 7E and Supplemental Figure 10A). The spleens of pI:pC-treated *Samd9l-Mut* mice had a markedly expanded red pulp with distortion of white pulp, possibly due to extramedullary hematopoiesis, which is commonly seen in mice in response to the diminished ability of BM to generate erythroid cells in stress conditions (Supplemental Figure 10, B and C) (27). We complemented these findings with flow cytometric assessment of erythroid maturation in both the spleen and PB, as previously reported (28). Treatment with pI:pC resulted in a significant increase in immature erythroid precursors in both spleen and PB and a decrease in the circulating mature erythroid cells in *Samd9l-Mut* mice (Supplemental Figure 10, D and E). In support of these data, RBCs and platelets were significantly reduced exclusively in the pI:pC-treated *Samd9l-Mut* mice, consistent with pancytopenia (Supplemental Figure 10F). Together, our data demonstrated that inflammation potentiates *Samd9l-Mut* phenotypes, in particular, pancytopenia with lymphopenia, corresponding to clinical observations in patients with *SAMD9L* mutations (13, 29–31).

### Samd9l-Mut mice recapitulate cellular phenotypes seen in patients.

**Figure 8:**
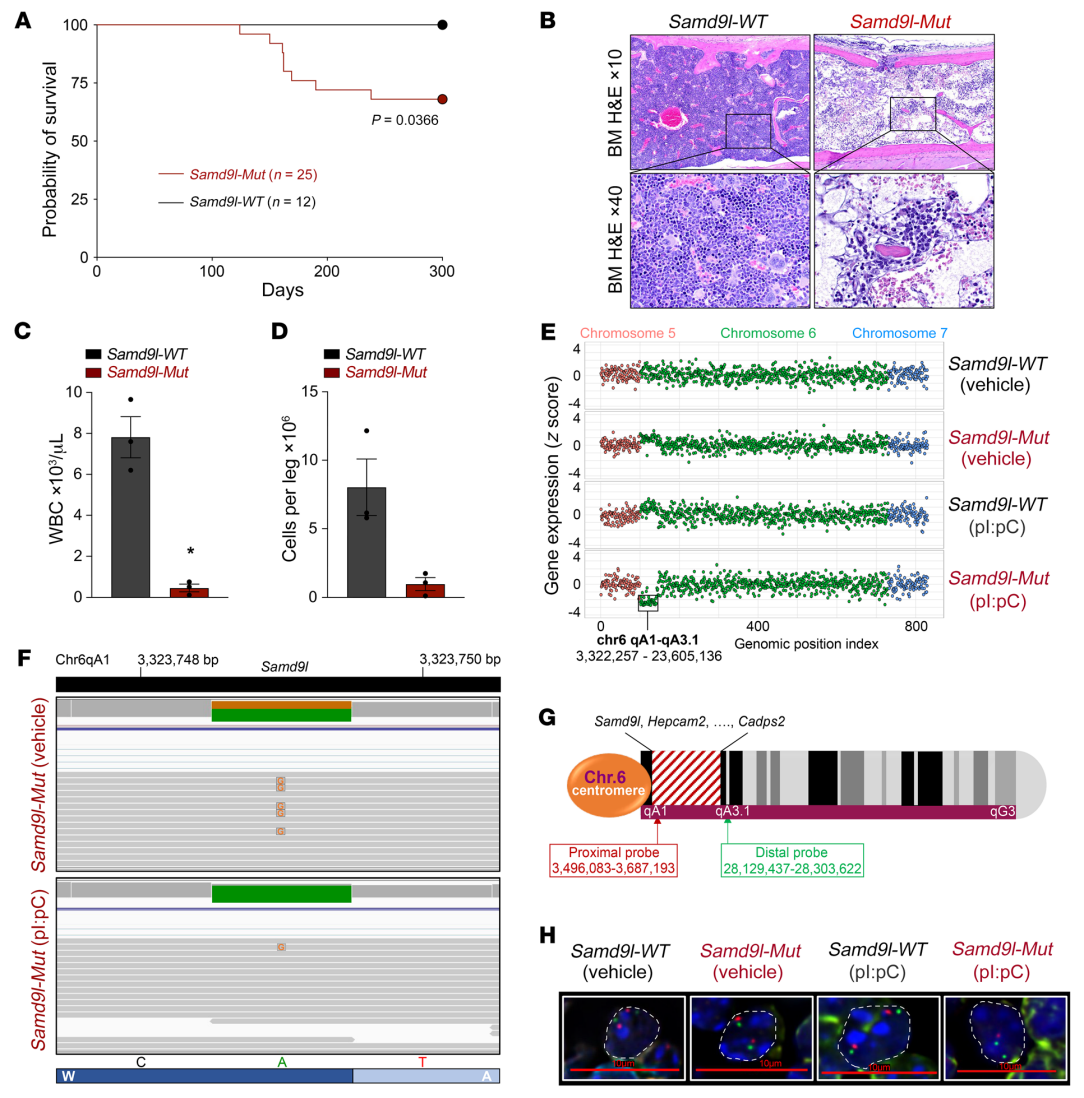
*Samd9l* mutation induces bone marrow hypocellularity and nonrandom chromosome 6 loss in vivo. (**A**) Survival analysis of *Samd9l-WT* (*n* = 12) and *Samd9l-Mut* mice (*n* = 25) over 300 days. Median survival was not reached by either group. Kaplan-Meier estimates were used, and significance was evaluated using the log-rank test. (**B**–**D**) Pathological assessment of *Samd9l-Mut* mice (died at 6 months) versus age-matched *Samd9l-WT* mice. (**B**) BM sections of *Samd9l-Mut* or *Samd9l-WT* mice (H&E; magnification, ×10 and ×40). (**C**) Peripheral blood WBC (*n* = 3). (**D**) Cell counts of viable BM cells per leg (*n* = 3). Wilcoxon’s rank-sum pairwise comparisons test was used for statistical analysis. *P < 0.05. Data shown as mean ± SEM. (**E**) Schematic of the affected region in chromosome 6 (chr6, qA1–qA3.1) in pI:pC-treated *Samd9l-Mut* mice relative to vehicle-treated *Samd9l-Mut* mice or *Samd9l-WT* mice treated with vehicle or pI:pC. The illustration shows the end of chr5, the entire chr6, and the start of chr7. The *y* axis shows the gene expression, and the *x* axis shows a genomic position index where each dot represents a gene and corresponds to its location. (**F**) Integrative Genomics Viewer (IGV; https://software.broadinstitute.org/software/igv/home) plot from RNA-seq analysis showing RNA transcripts of *Samd9l* wild-type allele (green) and *Samd9l* W1171R (A>G) heterozygous mutation (orange) in both *Samd9l-Mut* treated with vehicle (upper track) and *Samd9l-Mut* treated with pI:pC (lower track). (**G**) Illustration of mouse chr6 showing the affected region (red stripes) and the positions of the custom-designed FISH probes to detect the deleted region (proximal to *Samd9l* locus) or control nondeleted region (distal from *Samd9l* locus). (**H**) FISH analysis of spleens from *Samd9l-Mut* or *Samd9l-WT* mice treated with vehicle or pI:pC. FISH probes: proximal probe at chr6:3,496,083–3,687,193 (red) and distal probe at chr6:28,129,437–28,303,622 (green). Nuclei were stained with DAPI and are outlined by white dashed lines. Images were acquired on a Nikon C2 laser scanning confocal microscope (60×). Scale bar: 10 μm.

Finally, prolonged evaluation of these *Samd9l-Mut* mice demonstrated a phenotype mimicking BMF with incomplete penetrance, with approximately one-third of mice becoming symptomatic at the age of 6 months (Figure 8A). The moribund mice had a marked BM hypocellularity along with profound pancytopenia in PB (Figure 8, B–D, and Supplemental Figure 10G). The mice also demonstrated marked splenomegaly (Supplemental Figure 10H). Of note, the survivors did not have a change in BM cellularity, nor any acquired somatic revertant mutations in *Samd9l* that would provide a rescue mechanism indicated by targeted *Samd9l* sequencing, as observed in patients (10). Strikingly, however, we observed a significant downregulation of 42 genes (including *Samd9l*) in a specific locus on chr6 in 1 out of 3 pI:pC-treated *Samd9l-Mut* mice analyzed by RNA-seq (Supplemental Figure 11A). The affected region extended for approximately 20 megabase pairs along chr6 qA1–qA3.1 (chr6:3,322,257–23,605,136) and is syntenic to human chr7 (Figure 8E). Interestingly, this partial deletion in chr6 only occurs on the mutant allele, leaving an intact *Samd9l* wild-type allele (Figure 8F). This phenomenon was further investigated with fluorescence in situ hybridization (FISH) on splenic sections using custom probes to detect chr6:3,496,083–3,687,193 (proximal to *Samd9l* within the affected region) and chr6:28,129,437–28,303,622 (distal end of chr6 outside the affected region) loci (Figure 8G). We observed deletions at the proximal locus but not the distal locus in pI:pC-treated *Samd9l-Mut* mice (Figure 8H). As predicted, we also observed a deletion in an intermediate probe at chr6:22,116,691–22,428,747 (Supplemental Figure 11B). No deletions were observed in *Samd9l-WT* (treated with vehicle or pI:pC) or vehicle-treated *Samd9l-Mut* (Figure 8H). In total, we identified 4 mice with chr6 deletions within a cohort of *Samd9l-Mut* mice treated with pI:pC (*n* = 26) using either FISH and/or a validated qPCR screening analysis. The deletions were quantified by FISH in at least 3 mice and were observed in 70%, 53%, and 24% of the examined spleen cells, including those in the red pulp where myeloid cells are predominant.

## Discussion

Here we generated a mouse model to evaluate the in vivo hematopoietic impact of mutant *Samd9l* expression from the native locus. Our data demonstrated that heterozygous *Samd9l* mutation expression within hematopoietic cells impaired cell proliferation and self-renewal, which was potentiated by inflammation, leading to the induction of apoptosis, consistent with our previous ex vivo observations (18). Using a range of phenotypic and single-cell transcriptomic analyses, we found a decrease in WBC counts and significant alterations in the frequencies of hematopoietic compartments in the BM of *Samd9l*-mutant mice relative to wild-type or *Samd9l-KO* mice, including MPs and LT-HSCs. However, the mutant cells paradoxically lack stem cell self-renewal, as demonstrated by serial colony-forming unit cell (CFU-C) replating and competitive transplantations. These data suggest that *Samd9l*-mutant mice have elevated numbers of poorly functioning stem cells, and this increase may occur to compensate for the impaired steady-state hematopoiesis caused by the mutations. Collectively, these data show that in vivo expression of mutant *Samd9l*, in part induced by inflammatory stress, can impair HSC fitness and ultimately lead to BM hypocellularity, a hallmark of pediatric MDS and BMF syndromes.

Additionally, we demonstrated a distinct lymphopenia that was exacerbated with inflammation. We correlated this observation to the higher expression levels of *Samd9l* in mature B cells, potentially rendering them more sensitive to the effects of expression of the mutant allele. In fact, this is not completely surprising, as clinical reports have shown that lymphopenia in general, and particularly B cell ablations, are observed in BMF syndromes, including patients with mutant *SAMD9L* (29, 31). A recent report also demonstrated B lymphopenia in mice with the *Samd9l*-D764N mutation leading to phenotypes that mimic MIRAGE syndrome (32). Inflammation is known to compromise leukocyte production and affect B cell maturation and likely synergizes with mutant *Samd9l* expression in our model (33). We also observed a concomitant increase in relative percentages of myeloid precursors in *Samd9l-Mut* mice, especially after inflammation, a potential response to the associated lymphopenia, a process known as emergency granulopoiesis (34).

Inflammation resulting from viral infections is a potential mediator of BM disorders via suppression of hematopoiesis (35–37). In fact, *SAMD9* and *SAMD9L* are host restriction factors activated as a defensive mechanism in response to infection with viruses such as vaccinia, myxoma, and rhinovirus (38–40). Our data emphasize the profound impact of inflammatory stimuli on inducing mutant *Samd9l* expression from the endogenous locus, triggering exaggerated hematopoietic suppression and apoptosis. Our hypothesis, supported by our data, is that inflammatory pathways can be particularly harmful to patients with *SAMD9L* mutations and could accelerate BM suppression, potentially leading to mortality or adaptive responses, such as the outgrowth of clones with chromosomal deletions. This also raises an important question of whether microenvironmental inflammation in patients with mutant *SAMD9L* may contribute to the disease progression, which requires further evaluation.

Our in vivo model recapitulates many of the clinical manifestations of patients with *SAMD9L* mutations, including pancytopenia, BM hypocellularity, and incomplete clinical penetrance. Ribosome regulatory pathways were significantly reduced after stimulating with inflammation, which upregulates mutant *Samd9l* expression, leading to translation suppression reminiscent of our ex vivo overexpression model (18) and consistent with recent clinical observations (13, 29). Further, our model highlights the variable clinicopathological features in patients with germline *SAMD9* and *SAMD9L* mutations. In some patients, the mutant hematopoietic cells overcome the associated impairment by a potentially beneficial somatic-revertant mosaicism that restores normal hematopoiesis (via copy-number neutral loss of heterozygosity or loss-of-function mutations in *cis* with the germline allele). In contrast, other patients develop a clone with a potentially deleterious removal of the pathogenic allele on chr7, which can ultimately lead to MDS/AML (5, 8, 41, 42). In our mouse model, we did not observe *cis* revertant mutation acquisition. However, importantly, we observed a deletion of a specific region in mouse chr6 syntenic to human chr7 that includes common genes like *Samd9l*, *Hepacam2*, and others. Part of this genetic locus, including *Samd9l*, was also reported to modulate proinflammatory cytokines, including TNF-α and IFN-α, after viral infections (43). We concluded that the observed chr6 focal deletions in our mouse model mimics the monosomy 7 deletions seen in *SAMD9L* patients. To the best of our knowledge, our mouse model is the first to demonstrate this clinically relevant and rare phenomenon of chromosomal deletion affecting the *Samd9l* locus secondary to a deleterious *Samd9l* variant. Given that the chromosomal deletions affect the mutant allele, we postulate that this genetic haploinsufficiency is an adaptive response to release the stress caused by inflammation-induced *Samd9l* mutation expression. This concept is referred to as adaptation by aneuploidy (44).

Finally, our data exposed the TGF-β pathway as a potential therapeutic target in patients with *SAMD9L* mutations. Congruently, its upregulation is observed in similar BMF syndromes, such as Fanconi anemia and Shwachman-Diamond syndrome and is associated with impaired hematopoietic self-renewal (25, 26). Importantly, treatment with SD-208 rescued the defective human and mouse cells with *SAMD9L* mutations, consistent with previous observations in other BM disorder syndromes (25, 26). We propose TGF-β inhibition as a potential adjuvant therapy to further study as a protective measure against *SAMD9L*-mutation-associated hematopoietic impairment. In fact, a TGF-β inhibitor exhibited promising results in patients with low- and intermediate-risk MDS (45). Altogether, the demonstrated clinical relevance of our model establishes it as an outstanding reagent to study disease pathogenesis and identify potential therapeutic targets.

## Methods

### Animals.

*Samd9l-KO* mice were provided by Hiroaki Honda and Toshiya Inaba at Hiroshima University (19). We enlisted Ingenious Targeting Laboratory to generate the conditional mouse model with a *Samd9l*-W1171R mutation corresponding to the human W1180R mutation using embryonic stem cell–based gene targeting, as previously reported (46). The targeting vector was constructed by subcloning from a C57BL/6 BAC clone using a homologous recombination–based technique. CD45.1 and C57BL/6 mice were obtained from The Jackson Laboratory. Blood was collected via retroorbital bleeding and kept in EDTA-coated tubes. BM was collected by flushing the long bones with 2% FBS in PBS. Spleens were harvested, crushed, and strained through a 0.4 μm filter. Lineage depletion was done using the EasySep Mouse Hematopoietic Cell Isolation kit (STEMCELL Technologies).

### Flow cytometry.

Cell surface staining was performed using fluorescently labeled antibodies (Supplemental Table 2). For apoptosis assessment, cells were washed with annexin V binding buffer (Thermo Fisher Scientific) and stained with annexin V for 15 minutes followed by DAPI. Flow cytometry was done using an LSR FORTESSA II (BD Biosciences) and analyzed using FlowJo software (TreeStar).

### Colony-forming unit.

Lineage-depleted BM (3,000 cells) was cultured in MethoCult GF M3434 medium (STEMCELL Technologies) and incubated at 37°C with 5% CO_2_ for 7 days. For serial replating, colonies were counted and harvested by washing in PBS and 10,000 cells were recultured in MethoCult medium for an additional 7 days for 2 rounds.

### RNA-seq and scRNA-seq.

For RNA-seq, RNA was isolated from Lin^–^cKit^+^ sorted BM cells and sequenced on a NovaSeq 6000 (Illumina) as previously reported (18). scRNA-seq was performed on WBM or Lin^–^cKit^+^ sorted BM cells (*n* = 1 per group). Cells were prepared and quantified following the 10× Genomics protocol. Cells (4,000) were isolated for single-cell barcoding and 5′ GEX library preparation using the 10× Genomics Chromium Next-GEM Single Cell V(D)J Reagent Kit v1.1 following the manufacturer’s protocol. Libraries were pooled and sequenced on a NovaSeq 6000 using SP Reagent kit v1.5 (Illumina, 20028401).

### RNA-seq read, mapping, and data analysis.

Gene expression data were analyzed using the following guidelines. Reads from aligned bam files were assigned to genes and counted using HTSeq v0.11.2 (47) with the GENCODE mouse release 67 gene annotation. We generated the gene count matrix, and the log_2_CPM (counts per million) values were used for downstream analysis. We required that at least 2 samples (equal to the smallest group in the RNA cohort) should have 10 or more read counts per million reads sequenced in order to consider a gene as expressed. Differential gene expression analysis was done using R package Limma v3.32.10 (48). *P* values were adjusted by the Benjamini-Hochberg method to calculate false discovery rate (FDR). Genes with FDR less than 0.05 were considered significantly differentially expressed. Clustering and visualization were performed by R package heatmap v1.0.12 with Euclidian distance and Ward.D2 linkage. Pathway enrichment analysis was performed by R package clusterProfiler (v3.6.0). Rich Factor and other plots were created using R package ggplot 2 (v3.0.0). GSEA (49) was performed by GSEA v.1.0 using MSigDB genesets c2.all v7.4 for each mouse group (http://www.gsea-msigdb.org/).

### scRNA-seq barcoding, mapping, and data analysis.

The feature-barcode count data matrix for each sample was generated from raw sequence FASTQ files using Cell Ranger v3.0 (50) and the GRCm38 mouse reference genome. The output data from Cell Ranger was further analyzed in R environment (v4.0.2, RRID: SCR_001905) using R package Seurat (v3.2.1, RRID: SCR_007322) with default settings. Count data matrices in each experiment were combined using the merge function, followed by excluding cells with less than 250 or greater than 6,000 features, or greater than 5% mitochondrial transcripts. Feature counts for each cell were normalized to 1 × 10^4^ counts/cell and natural log transformed using the Normalize Data function. The dimensional reduction was performed by principal component analysis (PCA) using the top 500 variable genes identified by vst analysis using the Find Variable Features function, followed by Jack Straw analysis and UMAP (uniform manifold approximation and projection, RRID: SCR_018217) using the top 10 principal components. Clusters were identified by the Find Neighbors and Find Clusters functions (resolution = 0.5) and annotated by expression of canonical hematopoietic markers (21, 22) identified by the Find Markers function (min.pct = 0.25). DEGs were identified by R package Limma (48) (v3.32.10, RRID SCR_010943) using the top 5,000 variable genes, and *P* values were adjusted by the Benjamini-Hochberg method to calculate FDR. Genes with FDR less than 0.05 were regarded as significantly differentially expressed. Gene Ontology analysis of DEGs was performed by DAVID (The Database for Annotation, Visualization and Integrated Discovery, v6.8; RRID: SCR_001881) (51, 52). Data visualizations were performed by R package ggplot2 (v3.3.2, RRID: SCR_014601), pheatmap (v1.0.12, RRID: SCR_016418), and the generic plot function of the R environment.

### BM cytospins.

A total of 75,000 BM cells suspended in 200 μL of media were spun onto glass slides using a Wescor Aerospray Cytocentrifuge, dried for 10 minutes, fixed in Aerospray reagent-grade methanol, and stained with the Wescor Aerospray (modified Romanowsky) according to the manufacturer’s protocol.

### Histology and IHC.

Following euthanasia, the sternum and spleen were collected and fixed in 10% neutral buffered formalin, followed by decalcification of the sternum in 10% formic acid. The tissues were embedded in paraffin and 4-μm sections were stained with hematoxylin and eosin (H&E) or were used for IHC analysis. IHC was performed on formalin-fixed, paraffin-embedded (FFPE) tissues sectioned at 4 μm. All assay steps for GATA1, CD3, and MPO, including deparaffinization, rehydration, and epitope retrieval, were performed on the Ventana Discovery Ultra autostainer with Ventana Reaction Buffer (Ventana, 950-300) rinses between steps. All assay steps for B220/CD45R and Pax5 were performed on the Bond Max with Bond wash buffer (Leica, AR9590) rinses between steps. Antigen retrieval and incubation with the primary antibody were performed. Labeling was visualized with streptavidin conjugated to horseradish peroxidase (Thermo Fisher Scientific, TS-125-HR; 10 minutes) and substrate containing the chromagen DAB (Thermo Fisher Scientific, TA-125-HDX; 5 minutes).

### FISH.

Purified proximal region BAC DNA (RP24-374M7/6A/chr6:3,496,083–3,687,193 GRCm39) was labeled with red-dUTP (Alexa Fluor 594, Molecular Probes) using the Nick Translation DNA Labeling System 2.0 (Enzo). Purified distal region BAC DNA (RP23-324L12/6B/chr6:28,129,437–28,303,622 GRCm39) was labeled with green-dUTP (Alexa Fluor 488, Molecular Probes) using the Nick Translation DNA Labeling System 2.0. The FFPE spleen slides were heated at 60°C for 30 minutes, deparaffinized with limonene twice for 10 minutes each at room temperature, placed in ethanol 3 times for 2 minutes each at room temperature, air-dried, placed in 10% buffered formalin for 1 hour at room temperature, rinsed with H_2_O, placed in citrate buffer for 1 hour at 90°C, rinsed with H_2_O, placed in pepsin (8 mg/mL) in 0.1 HCL at 37°C for 6 to 8 minutes, rinsed with H_2_O, and then air-dried. For the hybridizations, the proximal region deletion (red) and distal region (green) probes were combined with sheared mouse Cot-1 DNA (Invitrogen) and hybridized to the treated slides in tDenHyb-2 solution (Insitus Biotechnologies). The probe and slides were co-denatured at 80°C for 10 minutes and incubated overnight at 37°C. The slides were then briefly washed in sodium phosphate/Igepal CA-630 (MilliporeSigma) and stained with DAPI (1 μg/mL) and images were captured at 0.15 μm plane spacing using a Nikon E800 microscope with Nikon NIS-Elements AR imaging software with 3D deconvolution. The camera and objective lens that were used were a Hamamatsu Orca Flash 4.0 camera and a 60× PlanApo N.A. 1.4 lens; 300 cells from each sample were analyzed.

### Data availability.

RNA-seq and scRNA-seq data were deposited into the NCBI Gene Expression Omnibus (GEO GSE190566 and GSE191147, respectively).

### Statistics.

Details about statistical comparisons are provided in each figure legend. For 2-group comparisons, Wilcoxon’s test was performed to test the distribution difference. Comparisons across more than 2 groups were performed using ANOVA models followed by Tukey-adjusted pairwise comparisons or by performing the Kruskal-Wallis test followed by pairwise Wilcoxon comparisons. Longitudinal trends were evaluated with mixed-effects longitudinal regression models with the R packages lmer4 and emmeans R package (lmer4) (53, 54). In one case, rank-based methods were used due to violation of the normality assumption. For survival data, Kaplan-Meier estimates were computed and plotted by genotype. The difference in survival distribution between genotypes was examined using an exact log-rank test. All the computations were done using R (55) and all *P* values are 2-sided.

### Study approval.

All animal studies including husbandry, breeding, and experimental procedures were performed in accordance with protocols approved by St. Jude Children’s Research Hospital Institutional Animal Care and Use Committee.

## Author contributions

All the authors have approved their authorship and gave input to the manuscript. SA and JMK conceptualized the study. SA, MET, and JMK developed the methodology. SA, MET, TW, EX, JRS, VV, MV, and CR conducted the investigation. SA, MET, MU, MPW, JM, and LJJ conducted formal analyses of the data. HW and SP provided statistical analyses. SA, MET, and JMK wrote the original draft of the manuscript. SA and MET generated the figures. MET and JMK acquired funding. SA and JMK supervised the study.

## Supplementary Material

Supplemental data

## Version 1 09/08/2022

In-Press Preview

## Version 2 11/01/2022

Electronic publication
