# Supplementary material for: Mutant Samd9l expression impairs hematopoiesis and induces bone marrow failure in mice
Source: J Clin Invest. 2022 Nov 1;132(21):e158869. doi: 10.1172/JCI158869 (PMC9621136; doi:10.1172/JCI158869)
Supplement: Supplemental data [file jci-132-158869-s124.pdf]

Mutant *Samd9l* expression impairs hematopoiesis and induces bone marrow failure in mice

Sherif Abdelhamed[1#], Melvin E. Thomas III[1#], Tamara Westover[1], Masayuki Umeda[1], Emily Xiong[1], Chandra Rolle[1], Michael P. Walsh[1], Huiyun Wu[2], Jason R. Schwartz[3], Virginia Valentine[4], Marcus Valentine[4], Stanley Pounds[2], Jing Ma[1], Laura J. Janke[1,5], Jeffery M. Klco[1$].

Supplemental Methods

Patient materials

Commercially available human cord blood-derived CD34+cells (Lonza, Switzerland) were cultured in human medium (StemSpan SFEM-II (StemCell Technologies, Canada) enriched with human cytokines (PeproTech, NJ) IL-6 (100ng/ml), FLT3 (100ng/ml), SCF (100ng/ml), TPO (100ng/ml), 1uM Stem Regenin-1, and 35nM UM171 (StemCell Technologies, Canada). Patient samples harboring *SAMD9L-S626L* mutations were obtained with informed consent using a protocol approved by the St. Jude Children's Research Hospital Institutional Review Board. Bone marrow aspirates were collected, mononucleated cells were isolated by FicollTM (GE Healthcare) and cryopreserved in the St. Jude Biorepository.

Intracellular flow cytometry

Cell surface staining was first performed using fluorescently labeled antibodies (**Table S2**). Cell cycle and protein synthesis were assessed by Click-iT™ EdU or Click-iT™ Plus O-propargyl-puromycin (OPP) assays, respectively (Invitrogen, CA). Cells were incubated with 10uM EdU for 2h or 10uM OPP for 30 minutes in RPMI (ThermoFisher, MA) with 15% FBS and supplemented with murine cytokines including interleukin-3, interleukin-6, SCF, Thrombopoietin, and Flt3-l (PeproTech, NJ) as previously reported (1, 2). After incubation, cells were fixed in 2% paraformaldehyde, permeabilized in 0.5% Saponin and Click-iT reactions were performed with the appropriate reagents. For intracellular phospho-SMAD2/3 staining, cells were fixed in 2% paraformaldehyde, permeabilized with 0.5% triton X-100, and stained with pSMAD2/3 antibody (BD Biosciences, CA). All data were analyzed using FlowJo software (TreeStar, OR).

Sanger DNA and Samd9l targeted sequencing

For Sagner sequencing, genomic DNA was harvested using Quick-DNA MiniPrep (Zymo Research). Using the indicated primers (**Table S2**), gDNA was used to amplify the *Samd9*l-KI construct. The product bands (~5kb) were gel-cleaned and sequenced using Janus liquid handling robotics system (Perkin Elmer), Veriti thermal cyclers for sample preparation (Applied Biosystems), and 3730xl DNA Analyzers (Applied Biosystems). For *Samd9*l targeted sequencing, fragments of interest were amplified (~5kb) using the indicated primers in table S2, and libraries were performed using Illumina Nextera XT part number FC-131-1096 and sequenced using NovaSeq 6000 S4 flowcell, paired end 100 cycle.

Western blot

Cells were harvested by washing several times with PBS and lysed in denaturation lysis buffer (50mM Tris-HCl pH8.0, 150mM NaCl, 4% SDS, 0.5% triton x-100, 10% glycerol, and 5% BME), heated at 99C for 5-10 minutes and briefly sonicated. Protein concentration was calculated by Bradford assay and 20ug total protein was loaded per sample onto a 4-20% gradient agarose gel. Immunoblotting was performed by transferring the gel to the PVDF membrane. Blots were then blocked with 5% non-fat milk in TBST (blocking buffer) for 2h at room temperature and stained with indicated primary antibodies (1:500 or 1:1000 dilution) in blocking buffer overnight at 4C with gentle rocking. Blots were washed 3 times with TBST for 5 minutes and stained with HRP-conjugated secondary antibodies (1:2000 dilution) for 2h at room temperature with gentle rocking. Blots were washed 3 times with TBST for 5 minutes and visualized using chemiluminescence.

Microscopy

HEK293T cells were plated on 22mm diameter poly-L-lysine coated coverslips (Neuvitro, WA) and treated with IFN-α (1000U) for 24h. These cells were washed twice with PBS, fixed with 4% paraformaldehyde for 15min, permeabilized in 0.3% triton X-100 for 10 minutes, and blocked with 5% rat serum for 1h. Cells were then stained with anti-SAMD9 or anti-SAMD9L primary antibodies (**Table S2**) for 2h at room temperature and washed with PBS. Cells were then stained with donkey anti-rabbit for 2h at room temperature, washed with PBS, and stained with DAPI for 5 minutes. Coverslips were mounted using ProLong Diamond Antifade (Invitrogen, CA). Images were acquired on a Nikon C2 laser

scanning confocal microscope using a 60X oil-objective lens controlled by NIS-Elements software (Nikon, Japan).

Supplemental Tables

Supplemental Table S1: quantification of the bone marrow smears.

Series	Population	*Samd9l-WT* Veh	*Samd9l-WT* pI:pC	*Samd9l-Mut* Veh	*Samd9l-Mut* pI:pC
Myeloid Series	Myeloblast	9.2	9.2	13.5	20.3
	Promyelocyte	0.2	4.7	1.1	3.7
	Myelocyte	25.6	18.2	31.0	40.4
	Metamyelocyte	11.8	18.7	18.7	18.1
	Neutrophil	17.2	16.3	24.4	3.5
	Monocytic	9.9	13.4	3.4	9.6
Lymphocyte Series	Lymphoblast	2.4	1.3	0.2	0.2
	Lymphocyte	23.7	18.2	7.6	4.1

Supplemental Table S2: reagents and resources.

REAGENT or RESOURCE	SOURCE	IDENTIFIER
**Antibodies**		
BV605 anti-mouse/human CD45R/B220	Biolegend	103244
PE/Cy7 anti-mouse CD3ε	Biolegend	100320
AF700 anti-mouse/human CD11b	Biolegend	101222
PerCP-Cy5.5 Ly-6A/E (Sca-1) Monoclonal Antibody (D7)	Thermo Fisher	45-5981-82
PE/Cy5 anti-mouse CD127 (IL-7Rα)	Biolegend	135016
PE/Cy7anti-mouse CD34	Biolegend	128618
AF700 anti-mouse CD48	Biolegend	103426
APC-ef780 c-Kit (2B8)	eBioscience	47-1171-82
BV605 anti-mouse CD150	Biolegend	115927
BV711 CD16/CD32	eBioscience	56-0161-82
PE anti-mouse CD3ε	Biolegend	100308
PE anti-mouse CD4	Biolegend	130310
PE anti-mouse/human CD45R/B220	Biolegend	103208
PE anti-mouse/human CD11b	Biolegend	101208
PE anti-mouse Ly-6G/Ly-6C (Gr-1)	Biolegend	108408
PE anti-mouse TER-119/Erythroid Cells	Biolegend	116208
PerCP-cy5.5 anti-mouse CD71	Biolegend	113816

AF647 anti-Smad2 (pS465/pS467)/Smad3 (pS423/pS425)	BD Biosciences	562696
APC AffiniPure F(ab')$_2$ Fragment Donkey Anti-Rabbit IgG	Jackson ImmunoResearch	711-136-152
LIVE/DEAD™ Fixable Aqua Dead Cell Stain Kit	Thermo Fisher	L34957
AF700 anti-mouse CD45.1	Biolegend	110724
eFluor 450 monoclonal Antibody (104) CD45.2	Thermo Fisher	48-0454-82
Polyclonal antibody to Glycophorin A (CD235a)	Cloud-Clone	PAB704Mu01
Anti-Gata1	Abcam	ab131456
Anti- Myeloperoxidase (MPO)	Dako	A0398
Anti-Mouse CD45R/B220 Clone RA3-6B2	PharMingen	553084
Recombinant monoclonal PAX5	Abcam	ab109443
Anti-CD3-ε	SantaCruz	sc-1127
Recombinant Anti-SAMD9	Abcam	ab180575
Rabbit Polyclonal anti-SAMD9L	Proteintech	25173-1-AP
Anti-GAPDH (14C10)	Cell Signaling	2118S
APC Annexin V	BD Biosciences	550474
**Biological samples**		
Human cord blood-derived CD34+cells	Lonza	2C-101
Patient samples with *SAMD9L-p.S626L* mutations	St. Jude Children's Research Hospital	https://www.stjude.org/
**Chemicals, peptides, and recombinant proteins**		
SD-208 TGF-βRI (ALK5) inhibitor	Selleckchem	S7624
Murine IL-3	PeproTech	213-13
Murine IL-6	PeproTech	216-16
Murine Flt-3 Ligand	PeproTech	250-31L
Murine SCF	PeproTech	250-03
Recombinant Mouse IFN-alpha	R&Dsystems	12100-1
Polyinosine-polycytidylic acid (pI:pC)	Invivogen	tlrl-pic-5
**Critical commercial assays**		
Click-iT™ EdU Cell Proliferation Kit	Thermofisher	C10337
MethoCult™ GF	StemCell Technologies	M3434
**Deposited data**		
RNA-seq	GEO	GSE190566
scRNA-seq	GEO	pending
**Experimental models: Organisms/strains**		
Samd9l-/- (Samd9l-KO)	(3)	N/A
B6.Samd9l(cKI	Ingenious Targeting Laboratory	N/A
B6.Cg-Tg(Vav1-cre)A2Kio/J (Vav1- Cre)	Jackson Laboratory	008610
B6.SJL-Ptprca Pepcb/BoyJ (Cd45.1)	Jackson Laboratory	002014
C57BL/6J	Jackson Laboratory	000664
**Oligonucleotides**		
Genotyping		
Vav1-Cre F: CAGGTTTTGGTGCACAGTCA	This paper	N/A
Vav1-Cre R: GGTGTTGTAGTTGTCCCCACT	This paper	N/A
Internal control F: CTAGGCCACAGAATTGAAAGATCT	This paper	N/A
Internal control R: GTAGGTGGAAATTCTAGCATCATCC	This paper	N/A
LOX1: TCC CGA TTT CCA CAC AGA TTA GTC	This paper	N/A
SEQ1: GCG TTT TAT CAG AAG TGC TGG ACC C	This paper	N/A

Sanger sequencing		
GFPWW1 F: CCGCATCGAGCTGAAGGGCATCGAC	This paper	N/A
mCh SQ1 F: AGACCGCCAAGCTGAAGGTGAC	This paper	N/A
SEQ1 R: GCGTTTTATCAGAAGTGCTGGACCC	This paper	N/A
Samd P3 F: TCTGGCCAAAAGGAAAGCACCTAAG	This paper	N/A
Samd9l SF 33: CAAAGACTGGACCAAAGA	This paper	N/A
Samd9l SF 404: AAAACATGTTAGGTGATGTGG	This paper	N/A
Samd9l SF 815: AAATCAGTGAAGCCAGGG	This paper	N/A
Samd9l SF 1205: GCTCACTTGATGAATCTTAC	This paper	N/A
Samd9l SF 1600: GTTTTGGTGGTGTTCCTCTT	This paper	N/A
Samd9l SF 2023: GAAGAACACTTTTATCGAGG	This paper	N/A
Samd9l SF 2396; AAGAGAACGCCTATATTCTG	This paper	N/A
Samd9l SF 2745: CCTGGCATTACTCAACTCTT	This paper	N/A
Samd9l SF 3093: TACAAGACAACGCAAGGAAC	This paper	N/A
Samd9l targeted sequencing		
WT *Samd9*l-GFP F: CAAAGACCCCAACGAGAAGC	This paper	N/A
Mutant *Samd9l*-mCherry F: GGACATCACCTCCCACAACG	This paper	N/A
Samd9l R: GGGCTAGAAAGAGTAAGTAC	This paper	N/A
**Software and algorithms**		
R package Limma version 3.32.10	(4)	SCR_010943
R package pheatmap version 1.0.12	NA	SCR_016418
R package ggplot 2 version 3.0.0 and 3.3.2	(5)	SCR_014601
GSEA verson 4.1.0	(6)	SCR_003199
R environment version 4.0.2	(7)	SCR_001905
R package Seurat version 3.2.1	(8)	SCR_007322
UMAP	(McInnes and Healy, 2018) <arXiv:1802.03426>	SCR_018217
DAVID (The Database for Annotation, Visualization and Integrated Discovery, version 6.8)	(9, 10)	SCR_001881
R package emmeans	(11)	SCR_018734
FlowJo version 10	TreeStar	N/A

Supplemental figures

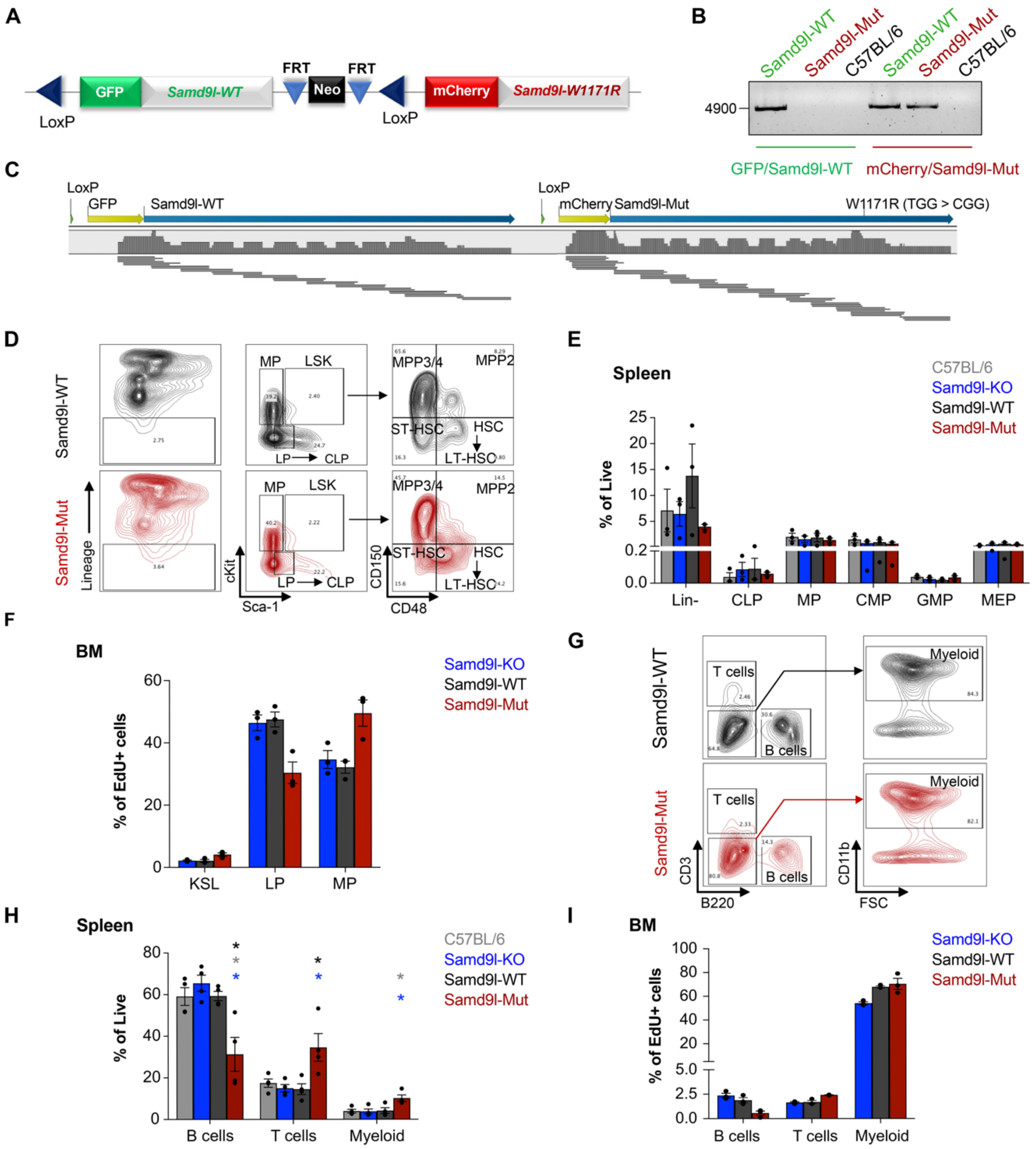

Figure S1: *Samd9l* mutant mice have altered hematopoietic differentiation and proliferation. A. Schematic showing the conditional insertion cassette. GFP-fused *Samd9l-WT* (inserted at exon 2) flanked by LoxP sites and a neomycin selection cassette flanked by FRT sites upstream of a stop codon and a mCherry-fused mutant *Samd9l* containing the W1171R mutation. The Neo cassette was removed before the Cre expression. After Cre recombination, the GFP-fused *Samd9l-WT* gene is removed and mCherry-fused *Samd9l-Mut* is expressed. **B**. Polymerase chain reaction (PCR) analysis of GFP-*Samd9l-WT* or mCherry-*Samd9l-Mut* fusions after recombination using *Samd9l*

targeted sequencing primers (Table S2). The gels show PCR products of 4900bp from *Samd9l-WT*, *Samd9l-Mut*, and C57BL/6 mice. Recombination was confirmed by deletion of the GFP-*Samd9l-WT* band in the *Samd9l-Mut* mouse. **C**. Graphical representation for the sequence coverage of the *Samd9l*-KI locus. Using overlapping primers (Table S2) insertion of the *Samd9l*-KI locus was confirmed by sanger sequencing. **D-E**. Flow cytometric analysis of C57BL/6, *Samd9l-KO*, *Samd9l-WT*, and *Samd9l-Mut* mice. (**D**) Gating strategy for HSPC populations. (**E**) Percentage of lymphoid or myeloid progenitors in the spleens (n=3). **F**. EdU incorporation assay by flow cytometry showing the contribution of HSPC and progenitor populations in the total proliferating cells from *Samd9l-KO*, *Samd9l-WT*, and *Samd9l-Mut* mice (n=3). **G-H**. Flow cytometric analysis of C57BL/6, *Samd9l-KO*, *Samd9l-WT*, and *Samd9l-Mut* mice. (**G**) Gating strategy for mature hematopoietic cells: B cells (B220+CD3-), T cells (B220-CD3+), and Myeloid (B220-CD3-CD11b+) (**H**) Percentages of mature cells in the spleens (n=4). **I**. EdU incorporation in the mature cells of *Samd9l-KO*, *Samd9l-WT*, and *Samd9l-Mut* mice (n=3). For statistical analysis, groups were initially compared by Kruskal-Wallis test, significant results were followed by pairwise comparisons with the Wilcoxon rank-sum test (p-values, *p<0.05, **p<0.01, ***p<0.001). Error bars indicate standard error (SEM) of the mean for biological replicates. For representation, C57BL/6 (grey), *Samd9l-KO* (blue), *Samd9l-WT* (black), and *Samd9l-Mut* (red).

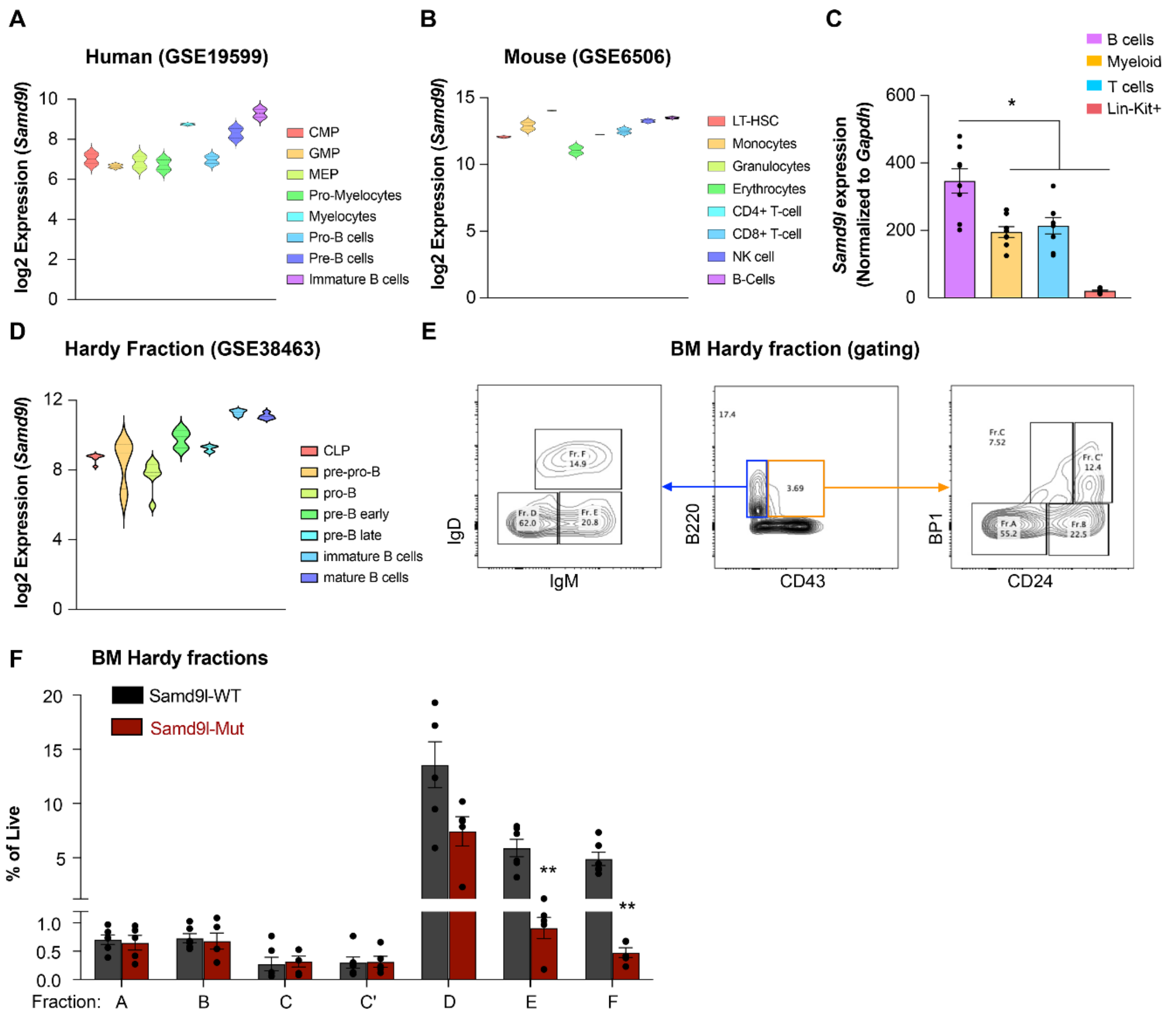

Figure S2: Higher expression of *Samd9l* in mature B cells may account for their increased sensitivity to the effects of the mutation.
A-B. Violin plots of the expression of *Samd9l* in different hematopoietic lineages from publicly available expression profiling arrays from human GSE19599 (**A**) and mouse GSE6506 (**B**) datasets. **C**. Expression of *Samd9l* from C57BL/6 sorted B cell (B220+, CD3e-), T cell (CD3e+, B220-), Myeloid (CD11b+, B220-, CD3e-), and Lin-Kit+ (cKit+, CD11b-, B220-, CD3e-) by qPCR (n=4). **D**. Violin plots showing the expression of *Samd9l* in different murine B cell lineages from publicly available gene expression data GSE38463. **E**. Flow cytometric gating strategy for Hardy fractions of B cells maturation stages as follows: Pre-pro-B (Fr.A) B220+ CD43+ BP1- CD24-; Pro-B (Fr.B) B220+ CD43+ BP1- CD24+; Pre-B (Fr.C-C') B220+ CD43+ BP1+ CD24+; Pre-B (Fr.D) B220+ CD43- IgM- IgD-; Immature B (Fr.E) B220+ CD43- IgM+ IgD-; and Mature B (Fr.F) B220+CD43- IgM+ IgD+. **F**. Flow cytometric analysis comparing the Hardy fractions in the BM of *Samd9l-WT* and *Samd9l-Mut* (n=6). For statistical analysis, groups were initially compared by Kruskal-Wallis test. Significant Kruskal-Wallis results were followed by pairwise comparisons with the Wilcoxon rank-sum test (p-values, *p<0.05, **p<0.01). Error bars indicate SEM of the mean for biological replicates.

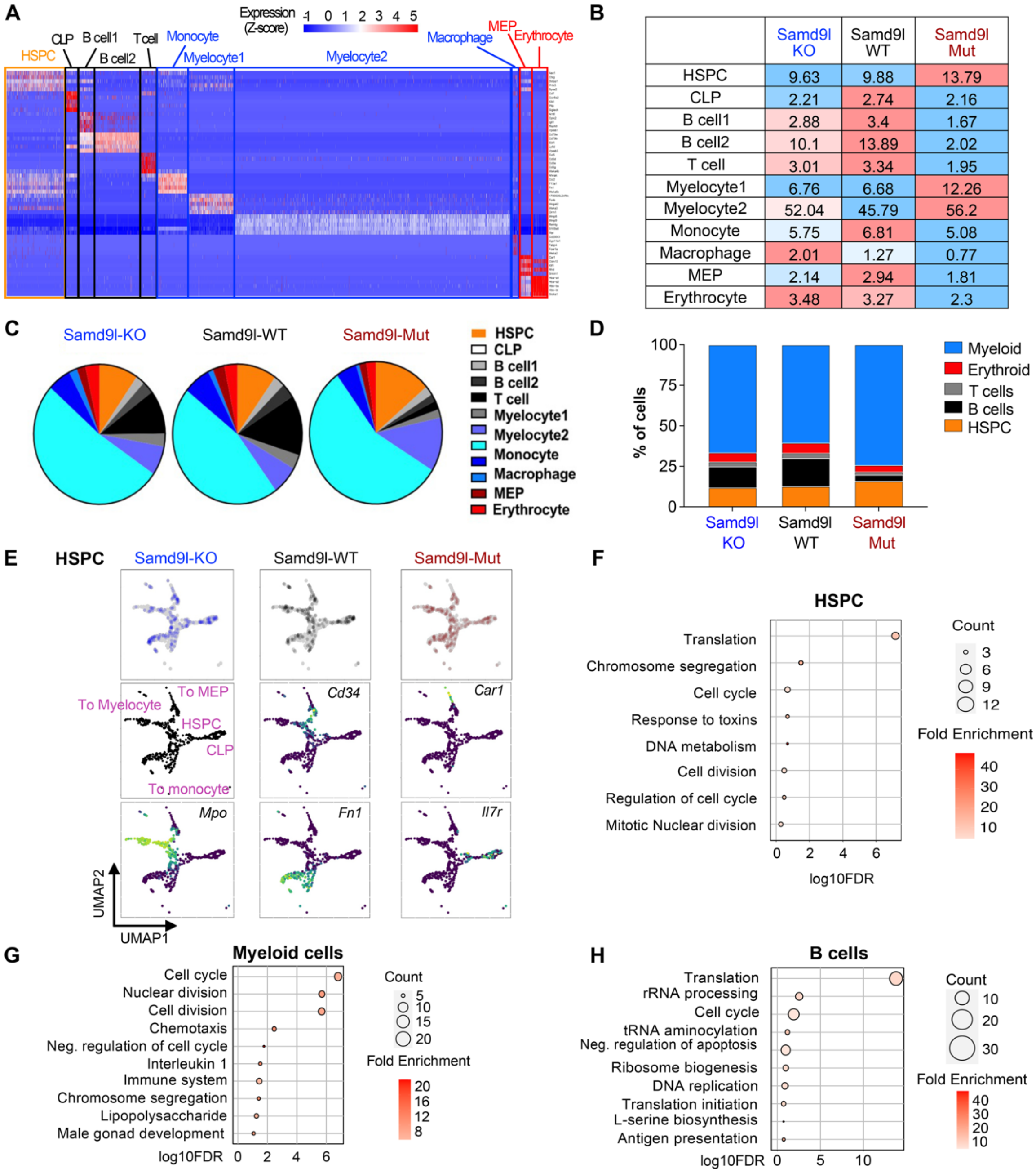

Figure S3: *Samd9l* mutant mice have a distinct profile of hematopoietic progenitors in single-cell analysis. A. A heatmap of single-cell RNA-seq data showing 11 clusters identified by well-established markers (12, 13). The top 5 uniquely expressed marker genes in each cluster were annotated. The color represents the z-scored expression level of each gene. **B-D**. A heatmap (**B**) or pie-charts (**C**) illustrating the proportion of the 11 clusters identified from single-cell RNA-seq analysis, (**D**) Bar graphs showing the proportion of the main populations in *Samd9l-KO*, *Samd9l-WT*, and *Samd9l-Mut* mice. In the heatmap, red or blue colors indicate high or low values among the groups, respectively, and numbers represent the percentages in total cells. **E**. UMAP plots demonstrating the differentiation trajectories of hematopoietic progenitors identified by

the indicated markers. **F**. A Rich factor plot showing the results of GO (Gene Ontology) term analysis for the DEGs between HSPC populations of *Samd9l-Mut* and *Samd9l-WT* mice. The horizontal axis represents logged FDR, and the color and size of circles represent the number and relative enrichment of genes in each GO term**. G.** A Rich factor plot showing the results of GO term analysis for the DEGs. The horizontal axis represents logged FDR, and the color and size of each dot represent numbers and relative enrichment of genes in each GO term. **H.** A Rich factor plot showing the results of GO term analysis for the DEGs between B cell populations of *Samd9l-Mut* mouse and *Samd9l-WT* mouse.

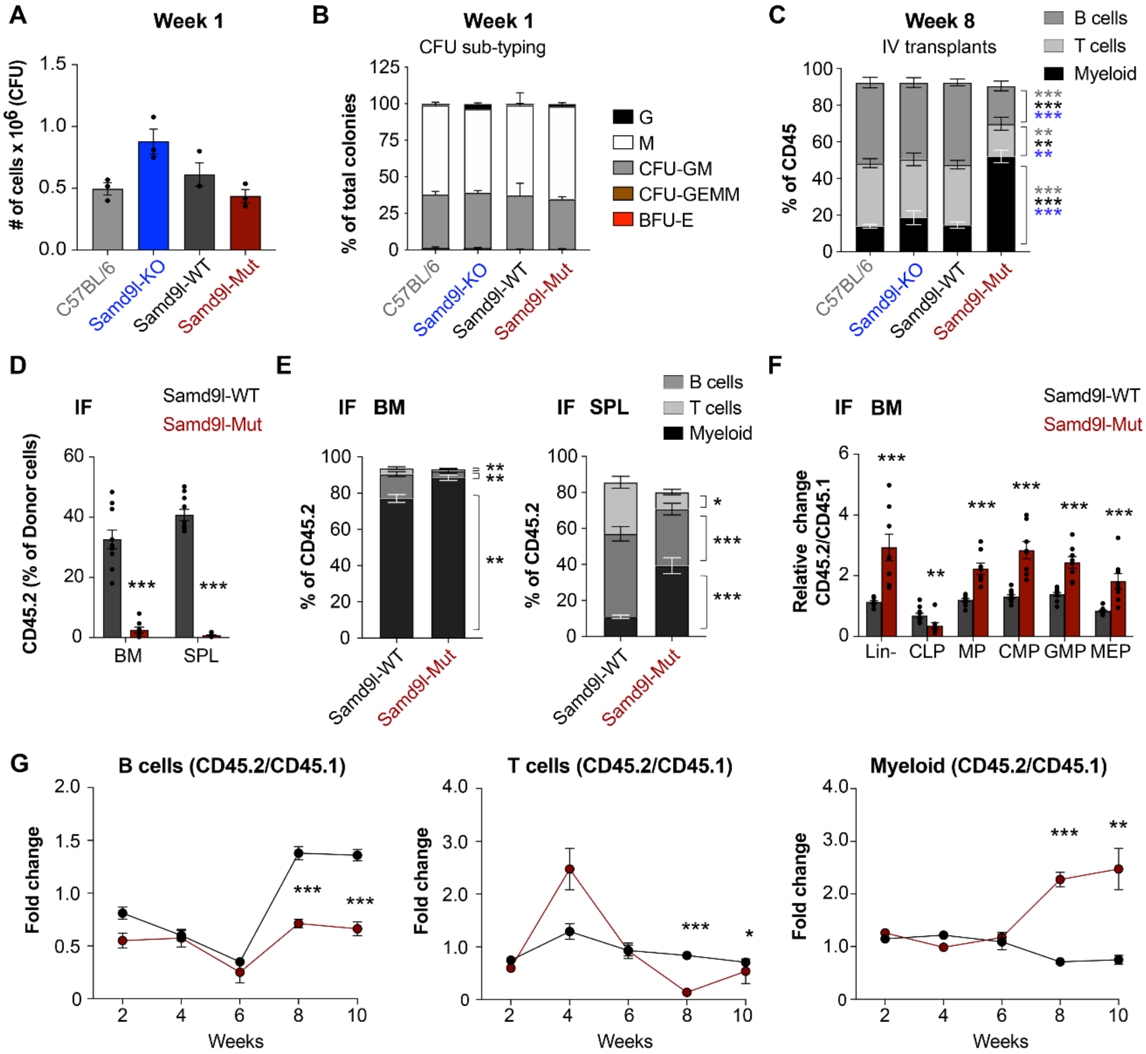

Figure S4: *Samd9l* mutant cells are less fit than normal counterparts. A. Number of cells per colony for C57BL/6 (grey), *Samd9l-KO* (blue), *Samd9l-WT* (black), and *Samd9l-Mut* (red) BM cells cultured for one week in methylcellulose (n=3). **B**. Relative distribution of the indicated colony subtypes in the tested groups (n=3). **C**. Mature cell populations (B, T, and Myeloid cells) in the peripheral blood of CD45.2 cells (C57BL/6, *Samd9l-KO*, *Samd9l-WT*, or *Samd9l-Mut*) from competitive transplants injected via tail-vein injections. Blood was collected at week 8 post-injection and assessed by flow cytometer (n=12). **D**. BM and spleen CD45.2 chimerism (*Samd9l-WT* or *Samd9l-Mut*) from competitive transplants via *intrafemoral* (I.F.) injections versus CD45.1 competitor cells (n=10). **E-G**. Flow cytometric analysis (n=10) showing the proportions of different mature cell populations in the (**E**) BM (left) and spleen (right); and (**F**) BM hematopoietic progenitors in CD45.2 cells (*Samd9l-WT* or *Samd9l-Mut*) from I.F. injected competitive transplants versus CD45.1. (**G**) Fold change of weekly peripheral blood percentages of B, T, or Myeloid cells in CD45.2 over CD45.1. For panels A-C, groups were initially compared by Kruskal-Wallis test, and if significant, followed by pairwise comparisons with Wilcoxon rank-sum test. For panel D-F, a two-way ANOVA global test was performed and followed by Wilcoxon rank-sum test between genotypes. For panel G, a longitudinal mixed effects

regression model was used for statistical analysis. A significant result was tested by evaluating the equality of effect at pre-specified time points between the two groups. (p-values, *p<0.05, **p<0.01, ***p<0.001). Error bars indicate SEM of the mean for biological replicates.

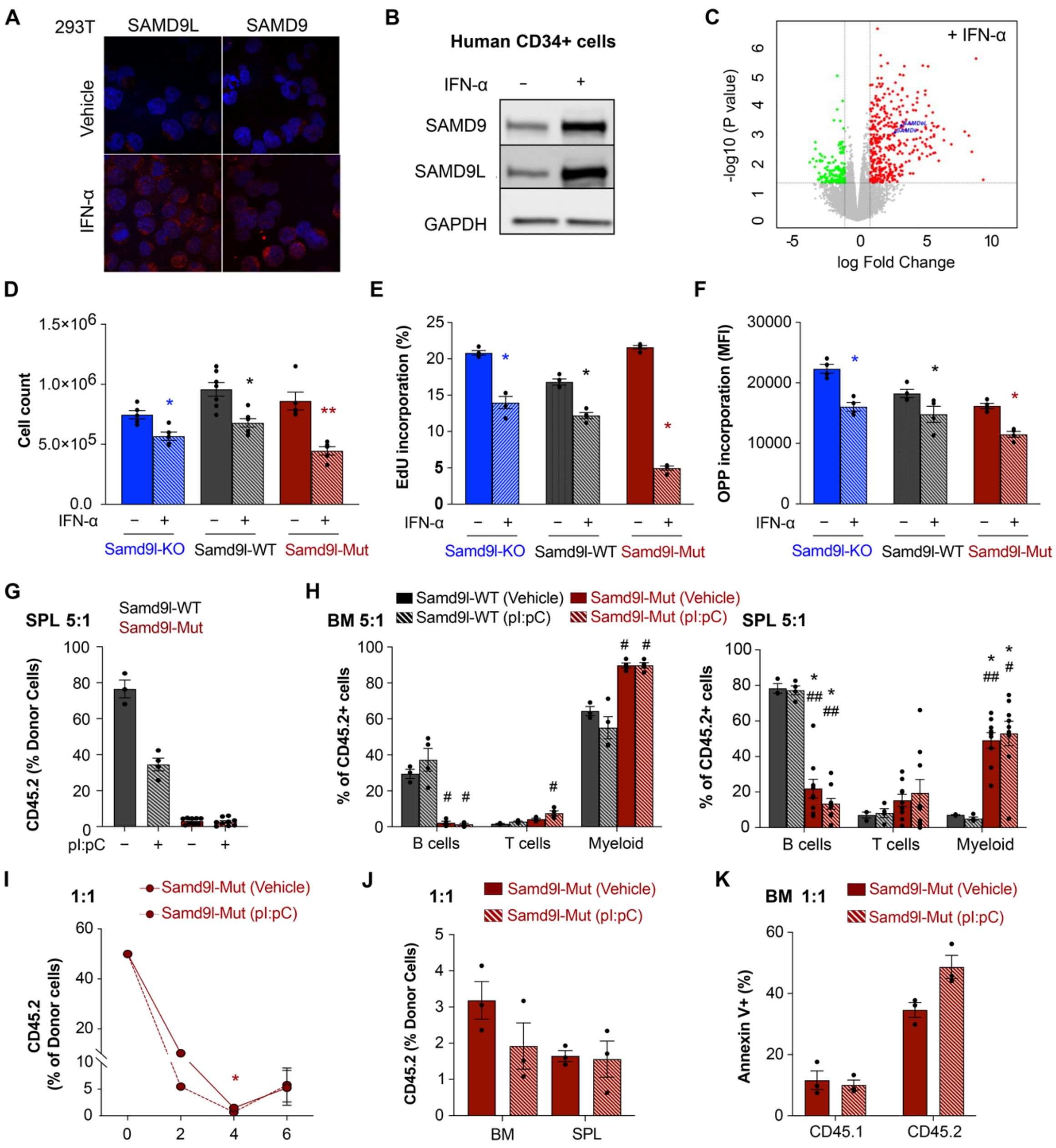

Figure S5: Inflammation regulates *Samd9l* expression and furthers the decrease in mutant cell fitness. A. Immunofluorescent microscopy of HEK293T treated with IFN-α or vehicle for 24h. Cells were labeled with anti-SAMD9 or anti-SAMD9L (red) and the DAPI nuclear stain (blue). Images were acquired on a Nikon C2 laser scanning confocal microscope (60x). **B**. SAMD9 or SAMD9L protein expression in human cord-blood $CD34^{+}$cells treated with vehicle or IFN-α (1000U) for 24h. **C**. Volcano plots of DEG in $hCD34^{+}$cells with and without IFN-α. *SAMD9* and *SAMD9L* genes were annotated. **D**. Cell count of BM cells treated twice with IFN-α (1000U) or vehicle for 48h from the indicated mice in Figure 4B (n=5). **E-F**. EdU (**E**) or O-propargyl-puromycin (OPP) incorporation (**F**) in BM of *Samd9l-KO*, *Samd9l-WT*, or *Samd9l-Mut* cells treated twice with IFN-α (1000U) or vehicle for 48h (n=4 per group). **G**. CD45.2 chimerism in the

spleens of *Samd9l-WT* and *Samd9l-Mut* treated with vehicle or pI:pC. The data shows the percentage of CD45.2 cells of the donor cells from 5:1 competitive transplants versus CD45.1 as described in Figure 4H. **H**. Mature cells percentage of CD45.2 cells in BM (left) or spleen (right) as described in Figure 4H. **I-J** Competitive transplants of *Samd9l-Mut* treated with or without pI:pC (CD45.2) versus CD45.1 (1:1 ratio). The data shows CD45.2 chimerism in (**I**) PB, (**J**) BM, and spleens. **K**. Annexin V percentage in CD45.1 or CD45.2 cells from BM of the 1:1 transplants. For panel I, a longitudinal mixed effects regression model was followed by evaluating the equality of effect at the pre-specified timepoints. For all other panels, pairwise comparisons Kruskal-Wallis test followed by multiple Wilcoxon rank-sum tests was used. Data show mean ±SEM. P-value: $^{\#,*}p<0.05$, $^{\#\#,**}p<0.01$, $^{\#\#\#,***}p<0.001$, color indicate the comparison group). For representation, *Samd9l-WT* (black), and *Samd9l-Mut* (red) (strips/dotted lines for pI:pC or IFN-α).

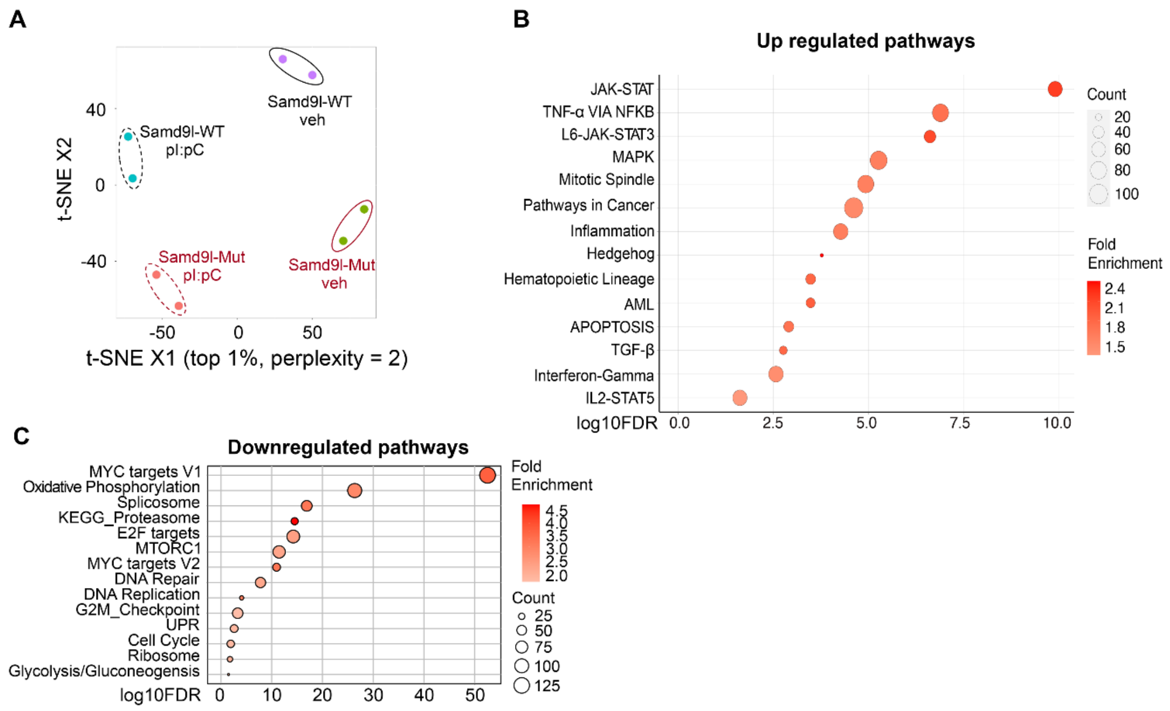

Figure S6: Transcriptional changes of LK cells after inflammation. A. A tSNE plot of vehicle- and pI:pC-treated *Samd9l-WT* and *Samd9l-Mut* LK cells (n=2 mice per condition).**B**. Pathways upregulated in pI:pC-treated relative to vehicle-treated *Samd9l-Mut* mice. The rich factor is determined by statistically significant genes divided by the total gene set, the size and color of dots represent gene count and fold enrichment, respectively. The position of the dots indicates the false discovery rate (FDR) significance for the indicated pathways. **C**. A plot of pathway enrichments of DEG from comparisons of vehicle or pI:pC treated *Samd9l-Mut* against *Samd9l-WT* groups and vice versa. The size of the circles is proportional to the significance (FDR) of the enrichment. The color is dependent on the rich factor of the analysis.

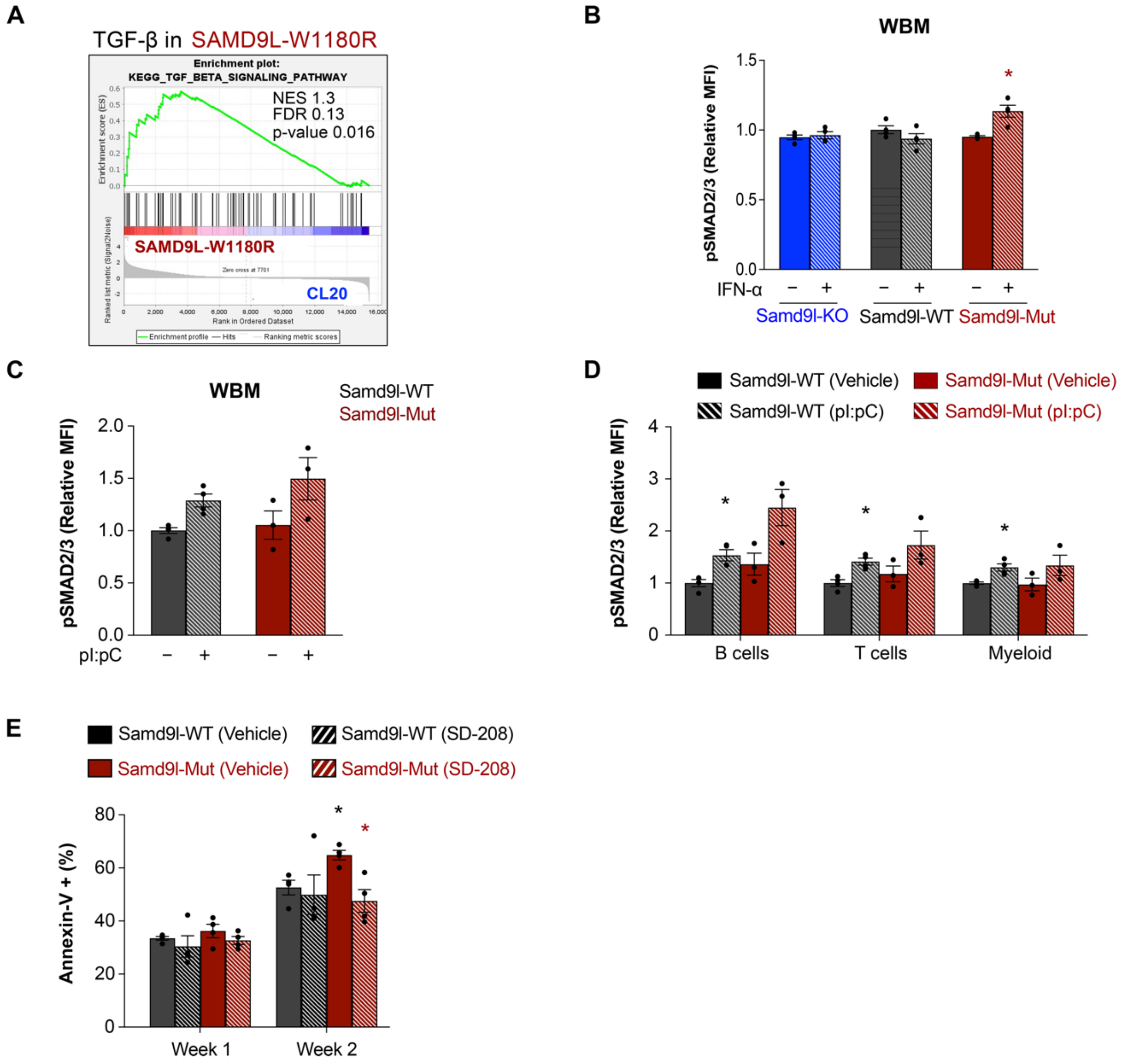

Figure S7: A. GSEA showing TGF-β pathway enrichment in hCD34+ cells overexpressing *SAMD9L-W1180R* relative to control hCD34+ cells transduced with empty vector (CL20). **B**. Intra-cellular phospho-SMAD2/3 expression in WBM form *Samd9l-KO*, *Samd9l-WT*, and *Samd9l-Mut* BM treated twice with IFN-α (1000U) or vehicle for 48h and assessed by flow (n=4). **C-D**. Intracellular phospho-SMAD2/3 expression in (**C**) WBM or (**D**) mature cells of *Samd9l-WT* (n=4), and *Samd9l-Mut* (n=3) BM treated with pI:pC or vehicle as described in Figure 5A. **E**. Annexin V percentage in cells after week 1 and week of CFU from *Samd9l-WT* or *Samd9l-Mut* treated with vehicle or SD-208 (n=4 per group). Statistics were measured by Kruskal-Wallis test followed by multiple Wilcoxon rank-sum tests for pairwise comparisons (p-values, *p<0.05, **p<0.01, ***p<0.001). Error bars indicate SEM of the mean for biological replicates. For representation, *Samd9l-KO* (blue), *Samd9l-WT* (black), and *Samd9l-Mut* (red). IFN-α or pI:pC (dotted lines) and vehicle (solid). Brown or grey colors were used for 1D11 mAb treated *Samd9l-Mut* or *Samd9l-WT* mice, respectively.

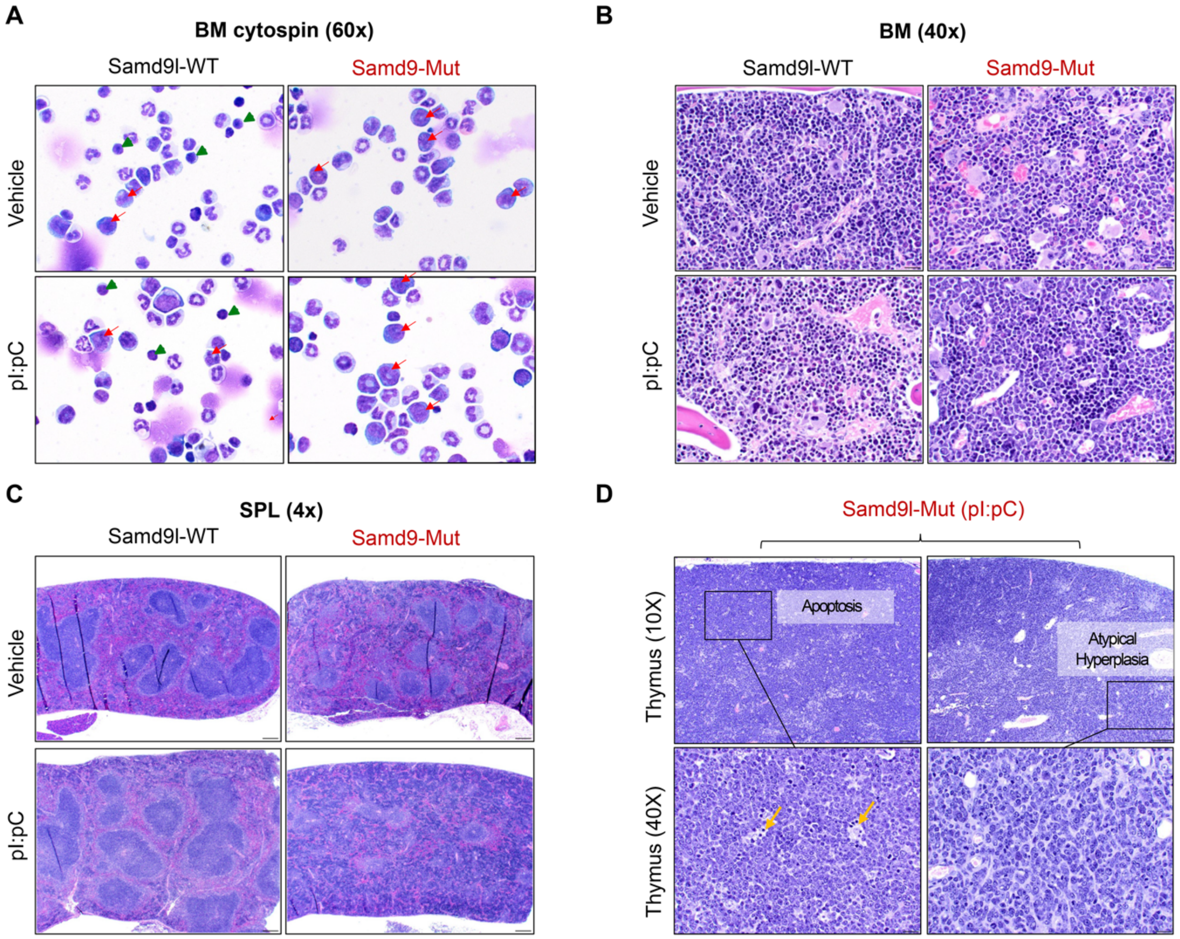

Figure S8: Inflammation exacerbates the pathogenesis of the *Samd9l* mutant mice. A. BM cytospins at 60x magnification stained with a modified Romanowsky stain. Red arrows = immature myeloid precursors and green arrowheads = lymphocytes. **B-C**. BM sections (H&E stain) at 40x magnification (**B**) and spleen at 4x magnification (**C**). **D**. Thymus sections (10X and 40X) from *Samd9l-Mut* mice treated with pI:pC and show either apoptosis in the cortex (left, n=2) or atypical hyperplasia (right, n=2).

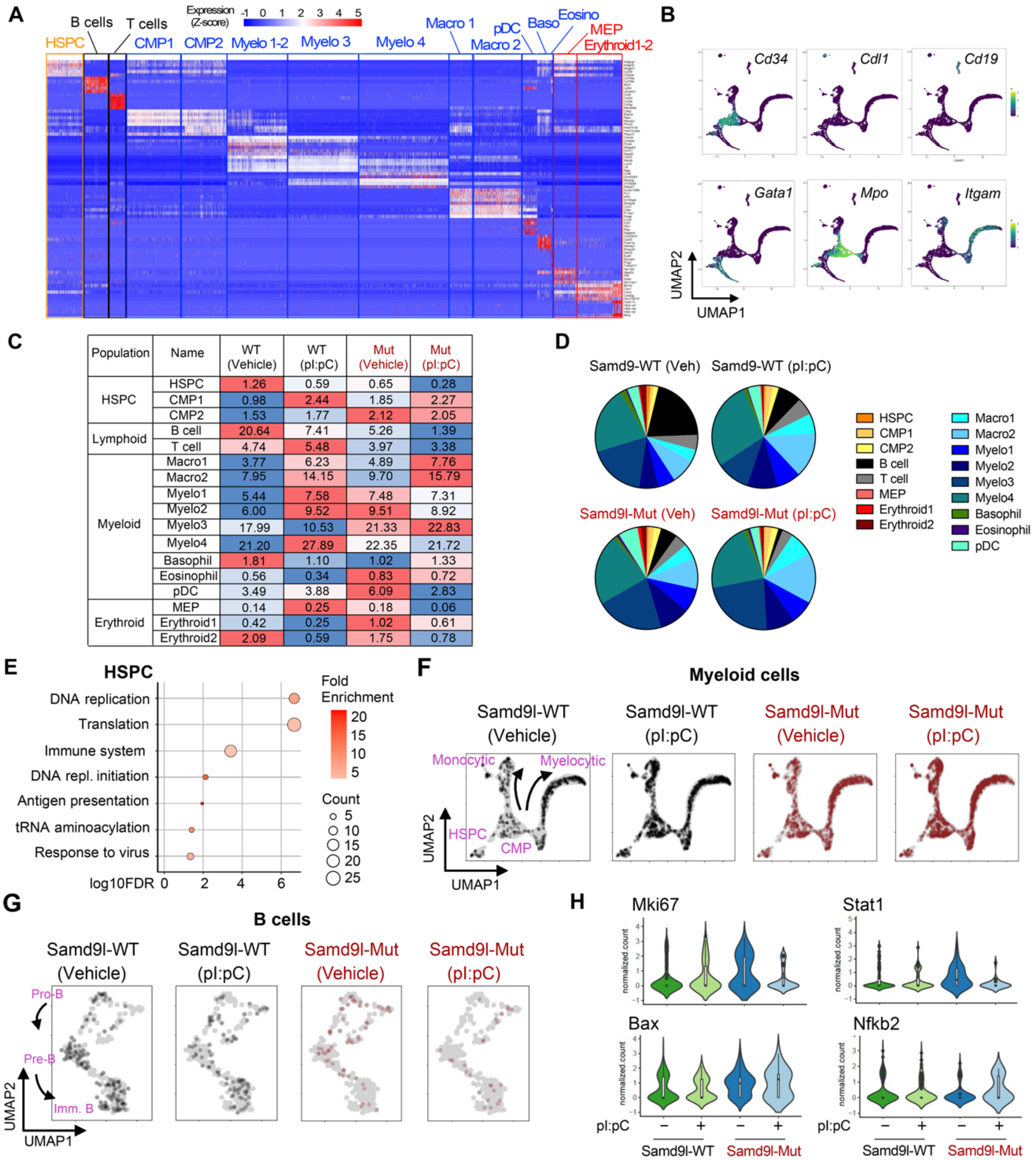

Population	Name	WT (Vehicle)	WT (pI:pC)	Mut (Vehicle)	Mut (pI:pC)
HSPC	HSPC	1.26	0.59	0.65	0.28
	CMP1	0.98	2.44	1.85	2.27
	CMP2	1.53	1.77	2.12	2.05
Lymphoid	B cell	20.64	7.41	5.26	1.39
	T cell	4.74	5.48	3.97	3.38
Myeloid	Macro1	3.77	6.23	4.89	7.76
	Macro2	7.95	14.15	9.70	15.79
	Myelo1	5.44	7.58	7.48	7.31
	Myelo2	6.00	9.52	9.51	8.92
	Myelo3	17.99	10.53	21.33	22.83
	Myelo4	21.20	27.89	22.35	21.72
	Basophil	1.81	1.10	1.02	1.33
	Eosinophil	0.56	0.34	0.83	0.72
	pDC	3.49	3.88	6.09	2.83
Erythroid	MEP	0.14	0.25	0.18	0.06
	Erythroid1	0.42	0.25	1.02	0.61
	Erythroid2	2.09	0.59	1.75	0.78

Figure S9: Inflammation further changes lineage composition in *Samd9l* mutant mice. A. A heatmap of the single-cell RNA-seq data from *Samd9l-WT* or *Samd9l-Mut* mice treated with either vehicle or pI:pC showing 17 clusters identified from the top 5 uniquely expressed marker genes in each cluster. The color represents the z-scored expression level of each gene. **B**. UMAP plots of representative markers for the main populations. The colors of each dot represent the normalized expression level of genes indicated above. **C.** A heatmap showing the proportion of the identified 17 clusters as well as the major 5 populations from each WBM sample. The red or blue colors indicate high or low values compared to the average of the groups, respectively, and numbers

represent the percentages in total cells. **D**. Pie charts showing the distribution of the identified 17 clusters in *Samd9l-WT* and *Samd9l-Mut* mice with or without pI:pC treatment. **E.** Rich factor plot showing the GO term analysis for the DEGs between HSPC populations of vehicle-treated and pI:pC-treated *Samd9l-Mut* mice. **F-G.** UMAP plots demonstrating the differentiation trajectories of Myeloid (**F**), or B cells (**G**) in *Samd9l-WT* and *Samd9l-Mut* mice treated with vehicle or pI:pC. **H**. Violin plots of the expression levels of differentially expressed genes between B cell populations of vehicle-treated or pI:pC-treated *Samd9l-Mut* mice. Representative genes involved in proliferation (Mki67 and Stat1), pro-apoptotic response (Bax), and inflammatory response (Nfkb2) are shown.

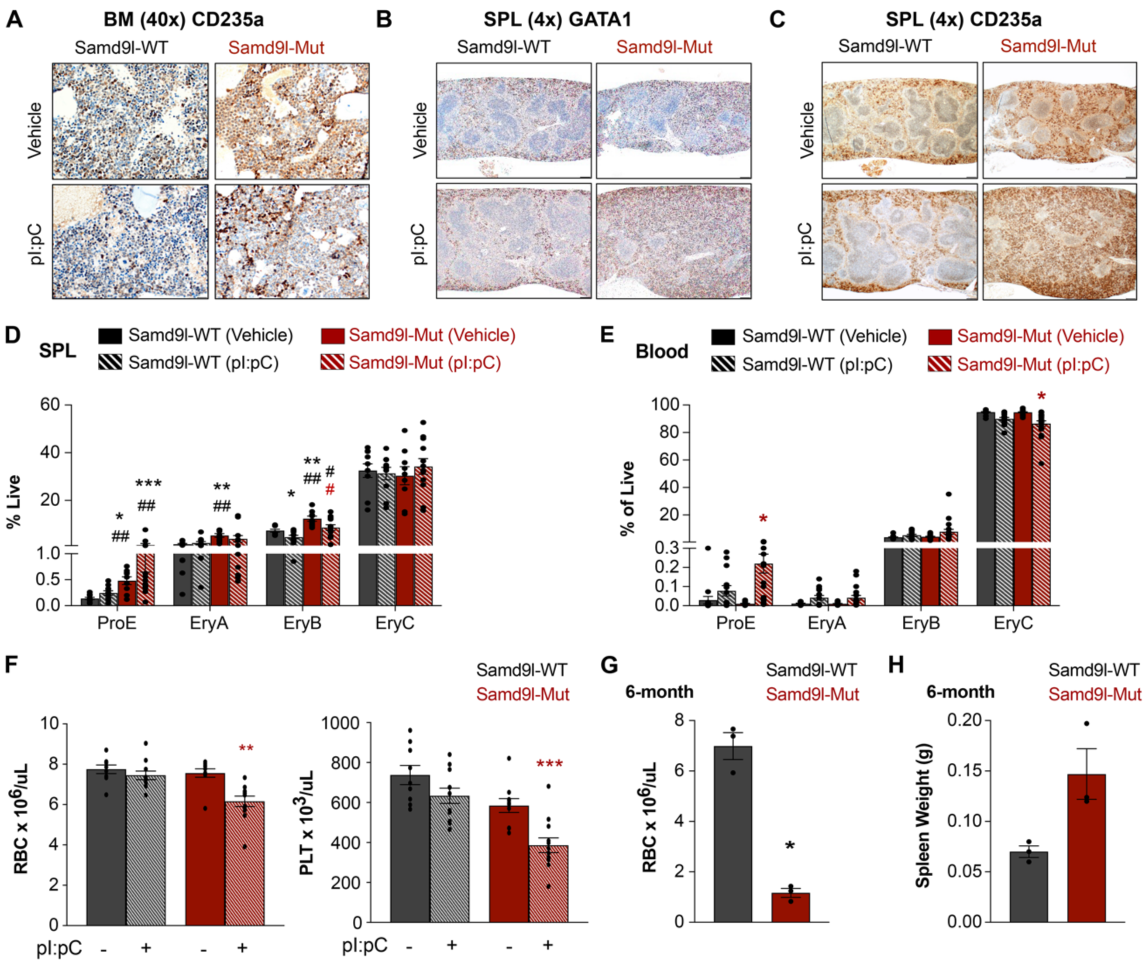

Figure S10: Inflammation exacerbates mutant *Samd9l* phenotypes. A. Cross sections of BM (40x magnification) of vehicle- and pI:pC-treated *Samd9l-WT* and *Samd9l-Mut* mice showing expression of CD235a. **B-C**. Cross section of spleens (4x magnification) from vehicle- and pI:pC-treated *Samd9l-WT* and *Samd9l-Mut* mice stained with (**B**) anti-Gata1 (**C**) anti-CD235a. **D-E**. Stages of erythroid maturation (ProE, EryA, EryB, and EryC) in the spleen (n=10) (**D**) or PB (**E**) of pI:pC or vehicle-treated *Samd9l-WT* (n=7) and *Samd9l-Mut* (n=8) mice assessed by flow cytometry after 4 weeks after pI:pC or vehicle treatment. **F**. CBC showing changes in red blood cells (RBC) and platelets (PLT) after 4 weeks post-pI:pC or vehicle treatment in *Samd9l-WT* and *Samd9l-Mut* mice (n=8). **G**. CBC (n=3) showing the RBC counts in lethargic *Samd9l-Mut* mice (red) relative to *Samd9l-WT* mice (black). **H**. Disease burden in the spleens of the lethargic mice showing spleen size in grams of the tested mice (n=3). For panels G and H, Wilcoxon test was performed to test the distribution difference between the 2 genotypes. For all other panels, Kruskal-Wallis test was performed and followed by multiple Wilcoxon rank-sum tests for pairwise comparisons. Error bars indicate SEM of the mean for biological replicates. For representation, *Samd9l-WT* (black), and *Samd9l-Mut* (red). (Dotted lines, p-values, $^{\#}p<0.05$, $^{\#\#}p<0.01$, $^{\#\#\#}p<0.001$, color indicate the comparison group) and vehicle (solid, p-values, $^{*}p<0.05$, $^{**}p<0.01$, $^{***}p<0.001$, color indicate the comparison group).

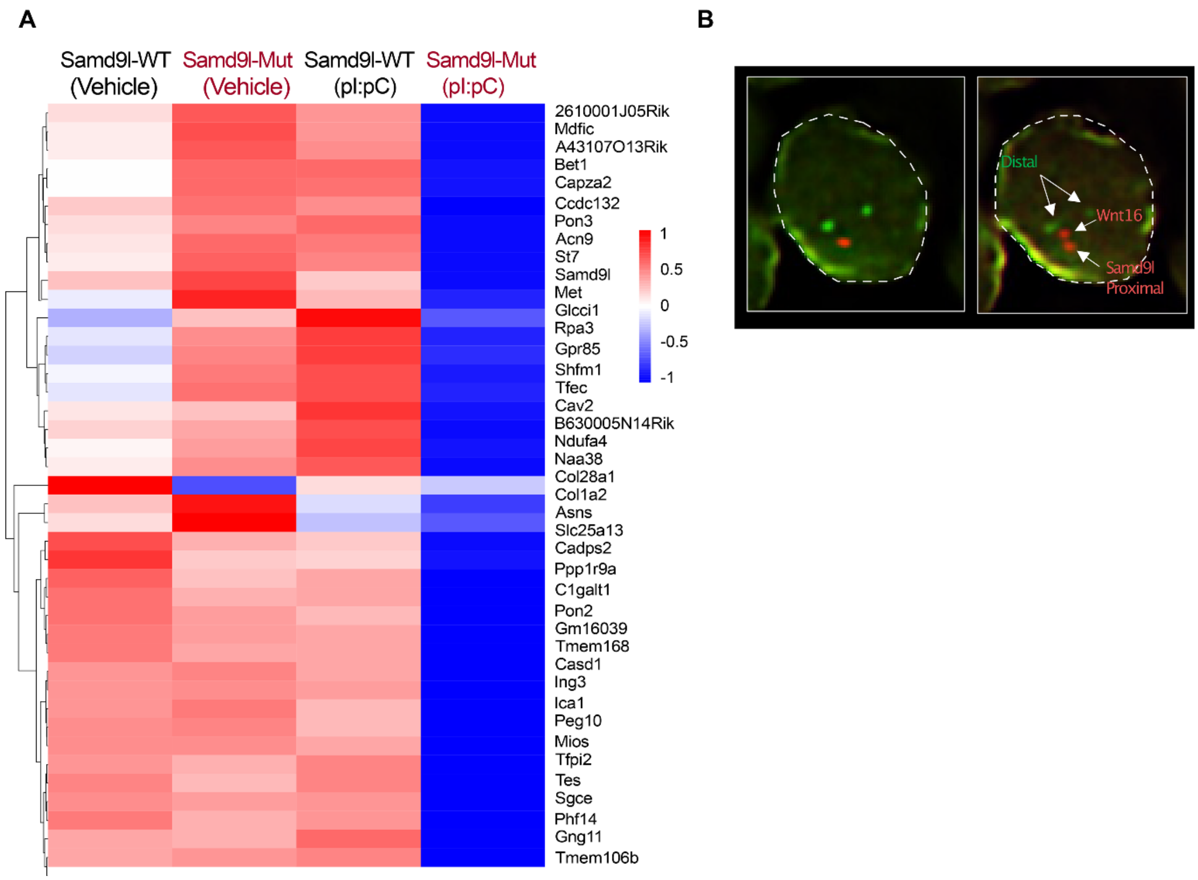

Figure S11: Inflammation induces non-random chromosome deletion in *Samd9l* mutant mouse. A. Heatmap of RNA-seq data from *Samd9l-WT* and *Samd9l-Mut* mice treated with vehicle or pI:pC (n=1 per group). The data shows the expression pattern of the expressed genes within the affected region at chromosome 6 (see also Figure 8E). **B**. FISH analysis of spleens from a *Samd9l-Mut* mouse treated with pI:pC showing the affected locations. Left image is showing the proximal at chr6:3,496,083-3,687,193 (red), and distal probe at chr6:28,129,437-28,303,622 (green). The right image is showing the same cell with an additional intermediate probe at chr6:22,116,691-22,428,747 (red, Wnt16). Nuclei were stained by DAPI and were outlined by white dashed lines. Images were captured using a Nikon E800 microscope with a 60X PlanApo objective lens. The imaging software used was Nikon NIS-Elements AR with 3D deconvolution.

References

1. Schwartz JR, Ma J, Lamprecht T, Walsh M, Wang S, Bryant V, et al. The genomic landscape of pediatric myelodysplastic syndromes. *Nat Commun.* 2017;8(1):1557.

2. Abdelhamed S, Butler JT, Doron B, Halse A, Nemecek E, Wilmarth PA, et al. Extracellular vesicles impose quiescence on residual hematopoietic stem cells in the leukemic niche. *EMBO Rep.* 2019;20(7):e47546.

3. Nagamachi A, Matsui H, Asou H, Ozaki Y, Aki D, Kanai A, et al. Haploinsufficiency of SAMD9L, an endosome fusion facilitator, causes myeloid malignancies in mice mimicking human diseases with monosomy 7. *Cancer Cell.* 2013;24(3):305-17.

4. Ritchie ME, Phipson B, Wu D, Hu Y, Law CW, Shi W, et al. limma powers differential expression analyses for RNA-sequencing and microarray studies. *Nucleic Acids Res.* 2015;43(7):e47.

5. Wickham H. ggplot2: Elegant Graphics for Data Analysis. Springer-Verlag New York. ISBN 978-3-319-24277-4, https://ggplot2.tidyverse.org. 2016.

6. Subramanian A, Tamayo P, Mootha VK, Mukherjee S, Ebert BL, Gillette MA, et al. Gene set enrichment analysis: a knowledge-based approach for interpreting genome-wide expression profiles. *Proc Natl Acad Sci U S A.* 2005;102(43):15545-50.

7. *R* Core Team R: A language and environment for statistical computing. R Foundation for Statistical Computing, Vienna, Austria. URL https://www.R-project.org/. 2021.

8. Satija R, Farrell JA, Gennert D, Schier AF, and Regev A. Spatial reconstruction of single-cell gene expression data. *Nature Biotechnology.* 2015;33(5):495-502.

9. Huang da W, Sherman BT, and Lempicki RA. Bioinformatics enrichment tools: paths toward the comprehensive functional analysis of large gene lists. *Nucleic Acids Res.* 2009;37(1):1-13.

10. Huang da W, Sherman BT, and Lempicki RA. Systematic and integrative analysis of large gene lists using DAVID bioinformatics resources. *Nat Protoc.* 2009;4(1):44-57.

11. Lenth RV. emmeans: Estimated Marginal Means, aka Least-Squares Means. R package version 1.6.2-1. https://CRAN.R-project.org/package=emmeans. 2021.

12. Giladi A, Paul F, Herzog Y, Lubling Y, Weiner A, Yofe I, et al. Single-cell characterization of haematopoietic progenitors and their trajectories in homeostasis and perturbed haematopoiesis. *Nat Cell Biol.* 2018;20(7):836-46.

13. Han X, Wang R, Zhou Y, Fei L, Sun H, Lai S, et al. Mapping the Mouse Cell Atlas by Microwell-Seq. *Cell.* 2018;172(5):1091-107.e17.
